# Tunable high-temperature itinerant antiferromagnetism in a van der Waals magnet

**DOI:** 10.1038/s41467-021-23122-y

**Published:** 2021-05-14

**Authors:** Junho Seo, Eun Su An, Taesu Park, Soo-Yoon Hwang, Gi-Yeop Kim, Kyung Song, Woo-suk Noh, J. Y. Kim, Gyu Seung Choi, Minhyuk Choi, Eunseok Oh, Kenji Watanabe, Takashi Taniguchi, J. -H. Park, Youn Jung Jo, Han Woong Yeom, Si-Young Choi, Ji Hoon Shim, Jun Sung Kim

**Affiliations:** 1 Center for Artificial Low Dimensional Electronic Systems, Institute for Basic Science (IBS), Pohang, Korea; 2 Department of Physics, Pohang University of Science and Technology (POSTECH), Pohang, Korea; 3 Department of Chemistry, Pohang University of Science and Technology (POSTECH), Pohang, Korea; 4 Department of Materials Science and Engineering, Pohang University of Science and Technology (POSTECH), Pohang, Korea; 5 Materials Modeling and Characterization Department, KIMS, Changwon, Korea; 6 MPPC-CPM, Max Planck POSTECH/Korea Research Initiative, Pohang, Korea; 7 Research Center for Functional Materials, National Institute for Materials Science, Tsukuba, Japan; 8 International Center for Materials Nanoarchitectonics, National Institute for Materials Science, Tsukuba, Japan; 9 Department of Physics, Kyungpook National University, Daegu, Korea

**Keywords:** Electronic devices, Magnetic properties and materials

## Abstract

Discovery of two dimensional (2D) magnets, showing intrinsic ferromagnetic (FM) or antiferromagnetic (AFM) orders, has accelerated development of novel 2D spintronics, in which all the key components are made of van der Waals (vdW) materials and their heterostructures. High-performing and energy-efficient spin functionalities have been proposed, often relying on current-driven manipulation and detection of the spin states. In this regard, metallic vdW magnets are expected to have several advantages over the widely-studied insulating counterparts, but have not been much explored due to the lack of suitable materials. Here, we report tunable itinerant ferro- and antiferromagnetism in Co-doped Fe_4_GeTe_2_ utilizing the vdW interlayer coupling, extremely sensitive to the material composition. This leads to high *T*_*N*_ antiferromagnetism of *T*_*N*_ ~ 226 K in a bulk and ~210 K in 8 nm-thick nanoflakes, together with tunable magnetic anisotropy. The resulting spin configurations and orientations are sensitively controlled by doping, magnetic field, and thickness, which are effectively read out by electrical conduction. These findings manifest strong merits of metallic vdW magnets as an active component of vdW spintronic applications.

## Abstract (web summary)

Metallic van der Waals magnets have considerable technological promise, due to their ability to be strongly coupled with electronic currents and integrated in two dimensional heterostructures. Here, Seo et al. demonstrate highly tunable itinerant antiferromagnetism in a van der Waals magnet.

## Introduction

Van der Waals (vdW) magnets^1–28^ offer an intrinsic magnetic multilayer system with various types of magnetic ground states^1–28^. Among them, the interlayer antiferromagnetism with an antiparallel spin configuration across the vdW gap is known to be effectively controlled by magnetic and electric fields, pressure, or doping^7–10^. This leads to various spin-functionalities, particularly for antiferromagnetic (AFM) spintronics, which have gained a lot of attraction recently due to their advantageous properties over ferromagnetic (FM) spintronics, including negligible stray field, robustness against magnetic perturbation, and ultrafast spin dynamics^29–32^. In contrast to the so-called synthetic AFM multilayers, where FM and non-magnetic (NM) layers are stacked alternately using the thin-film deposition techniques^29^, vdW antiferromagnets have a highly crystalline and atomically flat interface, free from interface roughness or chemical intermixing. This may lead to highly transparent and uniform magnetic proximity interaction for better spintronic performance in the magnetic multilayers, made of vdW antiferromagnets than synthetic AFM thin films.

One key issue for vdW material-based AFM spintronics is to identify suitable candidate materials. Most of the known vdW antiferromagnets are insulating^4–16^, and their interlayer magnetic coupling is mainly through superexchange-like interaction^11–13^. Thus, the modulation of interlayer coupling usually requires structural modification^9–14^ and is relatively difficult as compared to synthetic antiferromagnets with the interlayer Ruderman–Kittel–Kasuya–Yosida (RKKY) coupling^29^. Obviously, metallic vdW antiferromagnets^17–20^ can be a good alternative. The conduction electrons mediate the interlayer interaction, similar to synthetic antiferromagnets, which is expected to be strongly modulated by changing the composition or the interlayer distance. Furthermore, their longitudinal or transverse conductivities are sensitive to the spin configurations^33–38^, offering a direct probe to the spin states even for a few nanometer-thick crystals. Despite these merits, metallic vdW antiferromagnets are rare in nature, except a few recent examples of GdTe_3_ and MnBi_2_Te_4_ showing a low Neel temperature *T*_N_ <30 K^17–20^. Here, we show that an iron-based vdW material, Fe_4_GeTe_2_ with Co doping, hosts the interlayer AFM phase with *T*_N_ ~226 K in a bulk and ~210 K in 8-nm-thick nanoflakes. Its spin configuration is found to be effectively controlled and read out by conduction electrons, endowing Co-doped Fe_4_GeTe_2_ with a promising role in vdW-material-based spintronics.

## Results

### High-*T*_N_ antiferromagnetism in vdW structure

**Fig. 1:**
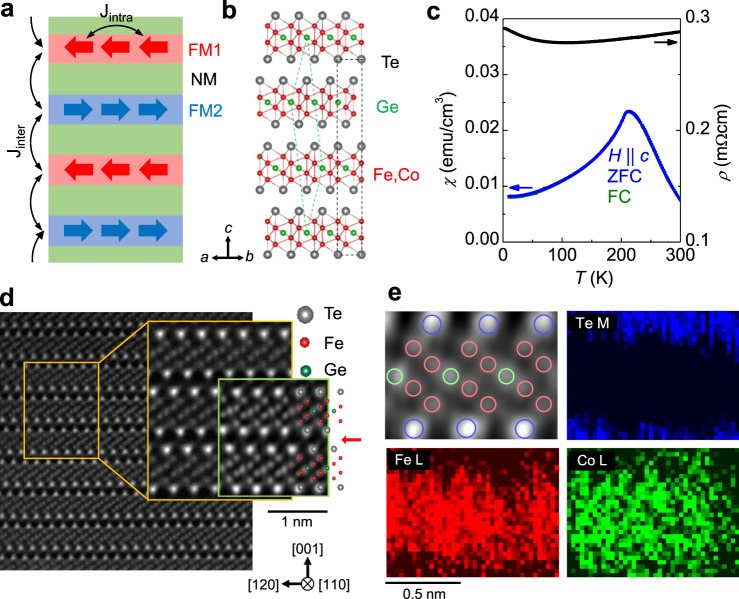
Crystal structure and antiferromagnetism of (Fe,Co)_4_GeTe_2_. **a**, **b** Schematic illustration of synthetic antiferromagnetic (AFM) multilayers and crystal structure of (Fe,Co)_4_GeTe_2_. In both cases, ferromagnetic (FM) layers with intralayer exchange coupling (*J*_intra_) are stacked alternately and coupled by interlayer coupling (*J*_inter_). **c** Temperature-dependent magnetic susceptibility *χ*(*T*) under a magnetic field *H* = 100 Oe along the *c*-axis and *ρ*(*T*) of (Fe_1 − *x*_Co_*x*_)_4_GeTe_2_ for *x* = 0.33. A clear cusp in *χ*(*T*), taken during the zero-field-cooling (ZFC) and field-cooling (FC) reveals the AFM transition at *T*_N_ ~ 210 K. **d** HAADF Scanning transmission electron microscopy (STEM) image of (Fe,Co)_4_GeTe_2_ (the first two ones) and Fe_4_GeTe_2_ (the last one) crystals along [1 1 0]. The STEM image with lower magnification shows that all layers are regularly arranged with a clear vdW gap (red arrow) and without stacking faults throughout whole areas. The comparison of STEM images with higher magnification of (Fe,Co)_4_GeTe_2_ (yellow box) and Fe_4_GeTe_2_ (green box) shows the almost identical atomic structure. **e** EELS intensity distributions of Te M, Fe L, and Co L edges within the monolayer of (Fe,Co)_4_GeTe_2_, indicating the possibilities of finding the corresponding atoms. A large intensity of Te M edge is found at the top and bottom of a monolayer as expected, whereas the intensity for Fe and Co atoms are uniformly distributed between Te layers. This infers that Co dopants successfully substitute Fe atoms as a solid solution.

We consider iron-based metallic vdW ferromagnets Fe_*n*_GeTe_2_ (*n* = 3–5) as a vdW analog of the synthetic multilayer systems (Fig. 1a)^21–28^. The first known member, Fe_3_GeTe_2_, is experimentally identified as a ferromagnet with *T*_c_ = 220 K^21–25^, but is theoretically predicted to be interlayer AFM via RKKY-like interaction^39^. The interlayer AFM coupling is, however, extremely fragile to the small amount of defects or dopants^39^, and the AFM phase is mostly inaccessible in real compounds. An alternative candidate is Fe_4_GeTe_2_, recently identified as a high *T*_c_ metallic ferromagnet with *T*_c_ = 270 K^26^. Because of the relatively thick slabs containing two Fe–Fe dumbbells (Fig. 1b), it has stronger intralayer FM coupling than Fe_3_GeTe_2_, together with the interlayer FM coupling, confirmed theoretically and experimentally^26^. By replacing 1/3 of Fe atoms with Co atoms, however, we found that it becomes AFM, as evidenced by its temperature-dependent susceptibility *χ*(*T*) under a magnetic field *H* = 100 Oe along the *c*-axis (Fig. 1c). Hereafter (Fe,Co)_4_GeTe_2_ denotes the AFM compound (Fe_1 − *x*_Co_*x*_)_4_GeTe_2_ (*x* = 0.33) unless the doping level *x* is otherwise specified. A clear cusp in *χ*(*T*), taken during the zero-field-cooling (ZFC) and field-cooling (FC), reveals AFM transition at the Neel temperature *T*_N_ = 210 K. The temperature-dependent resistivity *ρ*(*T*) is metallic with an upturn at low temperatures due to Kondo scattering (Fig. 1c and Supplementary Fig. S8). The conductivity of (Fe,Co)_4_GeTe_2_ is ~3 × 10^5^ Ω^−1^ m^−1^, which is comparable with that of the pristine Fe_4_GeTe_2_^26^ and in the bad metal regime (Supplementary Fig. S8). These characteristics make (Fe,Co)_4_GeTe_2_ a unique vdW AFM metal with the high *T*_N_ among vdW antiferromagnets (Supplementary Table S1).

The crystal structure of (Fe,Co)_4_GeTe_2_ is the same as Fe_4_GeTe_2_ in a rhombohedral structure (space group $R\overline{3}m$). Scanning transmission electron microscopy (STEM) image of (Fe,Co)_4_GeTe_2_ crystal visualizes the structural units of Fe–Fe dumbbells alternately above and below the plane of Ge atoms (Fig. 1d). These Fe–Fe–Ge–Fe–Fe layers are encapsulated with Te atoms, which shows a similar structural tendency to the pristine Fe_4_GeTe_2_ (the inset in Fig. 1d). A clear vdW gap between the layers is observed without any signature of stacking change or intercalated atoms throughout a wide region. These results imply that Co atoms are dominantly substituted to the Fe sites, not in a type of the interstitial sites. In Fig. 1e, electron energy loss spectroscopy (EELS) analysis visualizes the chemical information within a monolayer, which represents that Co atoms are homogeneously doped in all the Fe sites. Co doping results in the reduction of both the in-plane (*a* = 4.08 ± 0.04 Å) and out-of-plane lattice parameters (*c* = 29.10 ± 0.28 Å), which are deducted from the selected area diffraction pattern (SADP) analysis, in good agreement with X-ray diffraction results (Supplementary Fig. S1).

### Doping-dependent evolution of magnetic phases

**Fig. 2:**
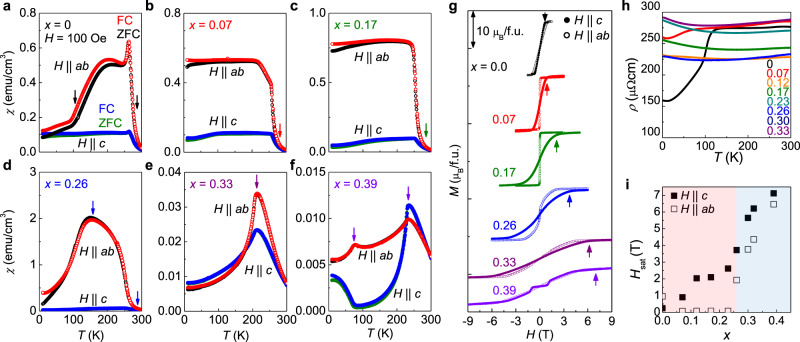
Doping-dependent magnetic phase diagram of (Fe_1 − *x*_Co_*x*_)_4_GeTe_2_. **a**–**f** Temperature-dependent magnetic susceptibility *χ*(*T*) of (Fe_1 − *x*_Co_*x*_)_4_GeTe_2_ (0.0 ≤ *x* ≤ 0.39), taken during zero-field cooling (ZFC) and field-cooling (FC) under *H* = 100 Oe with both magnetic field orientations, *H*∥*c* (solid) and *H*∥*ab* (open). **g** Magnetization *M*(*H*) as a function of magnetic field for (Fe_1 − *x*_Co_*x*_)_4_GeTe_2_ (0 ≤ *x* ≤ 0.39) single crystals, for different field orientations, *H*∥*c* (solid) and *H*∥*ab* (open). All the *M*(*H*) curves were taken at *T* = 10 K, except those for *x* = 0.39 taken at *T* = 100 K. **h** Temperature-dependent in-plane resistivity *ρ*(*T*) showing a metallic behavior. **i** The saturation fields *H*_sat_ for *H*∥*c* (solid) and *H*∥*ab* (open) as a function of Co doping *x*.

**Fig. 3:**
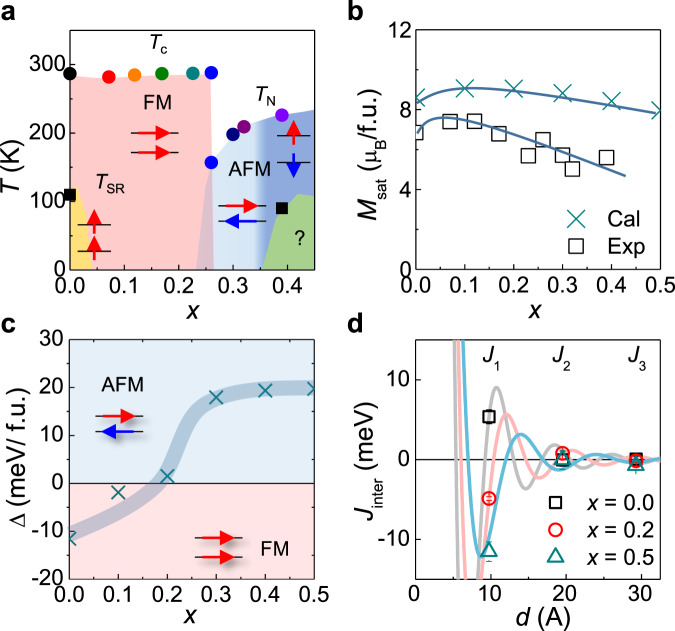
First-principles calculations of (Fe_1 − *x*_Co_*x*_)_4_GeTe_2_. **a** Phase diagram of spin states for (Fe_1 − *x*_Co_*x*_)_4_GeTe_2_ with Curie (*T*_C_) and Neel (*T*_N_) temperatures. Four different spin states in terms of interlayer spin configurations (FM and AFM) and spin orientations (easy-axis and easy-plane) are stabilized depending on doping level and temperature. The unknown magnetic phase is found for *x* = 0.39 below *T* = 90 K. The schematic illustration of spin configurations is shown in the inset. **b** The saturation magnetization *M*_sat_ (square symbol) and the calculated *M*_sat_ (cross symbol) as a function of Co doping *x*. **c** Doping-dependent total energy difference Δ between the FM and the A-type AFM phases. The FM-to-AFM transition occurs at the critical doping level *x*_c_ ~ 0.2, in good agreement with experiments. **d** Calculated interlayer exchange interaction *J*_inter_ with different layer distance (*d*) for *x* = 0, 0.2, and 0.5. The oscillatory dependence of *J*_inter_(*d*), captured by the RKKY model, is systematically changed with Co doping. The errors in the experimental data are smaller than the size of the points.

Having established the high-*T*_N_ AFM phase in (Fe,Co)_4_GeTe_2_, we focus on the systematic changes of the magnetic and electrical properties of (Fe_1 − *x*_Co_*x*_)_4_GeTe_2_ single crystals with a variation of Co doping (0 ≤ *x* ≤ 0.39). For *x* = 0, the FM transition with in-plane alignment of magnetic moments occurs at *T*_c_ = 270 K, which is followed by the spin-reorientation transition to the out-of-plane alignment at *T*_SR_ = 110 K (Fig. 2a)^26^. Co doping quickly suppresses the spin-reorientation transition, while keeping the FM transition almost intact with a nearly constant *T*_c_ for *x* ≤ 0.23 (Fig. 2b, c and Supplementary Figs. S2 and S3). Upon further Co doping, however, the AFM order develops from *T*_N_ = 155 K, well below *T*_c_ for *x* = 0.26, and eventually becomes dominant with high *T*_N_ up to 226 K for *x* = 0.39 (Fig. 2d–f). The negligible bifurcation between *χ*(*T*) curves taken during ZFC and FC is consistent with the long-range AFM phase. The saturation magnetization *M*_sat_ monotonically decreases with Co doping from 7.1*μ*_B_/f.u. (*x* = 0) to 5.5*μ*_B_/f.u. (*x* = 0.39) due to the magnetic dilution effect (Fig. 2g). Concomitantly, the out-of-plane saturation field ${H}_{{\rm{sat}}}^{c}$ gradually increases with Co doping in the FM phases for *x* < 0.26. This is consistent with the changes of the magnetic anisotropic energy (*K*) from the easy-axis to the easy-plane types, following ${H}_{{\rm{sat}}}^{c} = 2K/M_{\mathrm{sat}}$ (Fig. 2i). Entering the AFM phase, however, ${H}_{{\rm{sat}}}^{c}$ is determined by the AFM coupling *J* as described by ${H}_{{\rm{sat}}}^{c}$ ≈ 2*J*/*M*_sat_ and thus suddenly enhanced up to ~6 T for *x* = 0.39. In the AFM phase, the spin-flop transition is observed for *H*∥*ab* at *x* = 0.33, but *H*∥*c* at *x* = 0.39 (Fig. 2g and Supplementary Fig. S6), indicating the easy-plane and the easy-axis type spin alignments, respectively. For *x* = 0.39, we found that another magnetic transition occurs to the unknown phase below *T* = 90 K (Fig. 2f and Supplementary Fig. S2). The resulting phase diagram is summarized in Fig. 3a, which manifests that the magnetic configuration and also the magnetic anisotropy of (Fe_1 − *x*_Co_*x*_)_4_GeTe_2_ are highly sensitive to Co doping.

The first-principles calculations consistently predict the FM-to-AFM phase transition with Co doping. Total energy calculations for the FM and various AFM phases confirm that the FM phase is initially stable at low *x*, but eventually becomes unstable against the AFM phase at high *x*. The most stable AFM phase is the so-called A-type, in which all the spin moments in the whole slab of (Fe,Co)_4_GeTe_2_ are ferromagnetically aligned, but across the vdW gap, they are antiferromagnetically coupled. This is consistent with the positive Curie–Weiss temperature from the inverse susceptibility in the AFM phase, suggesting the dominant FM interaction within the layers (Supplementary Fig. S3). Furthermore, from resonant soft X-ray scattering experiments for Fe L3-edge, we observed the magnetic Bragg peak at *q* = (0 0 3/2) developed below *T*_N_ for the crystal with *x* = 0.33. This confirms the A-type AFM structure, as predicted by calculations (Supplementary Fig. S7). Figure 3c shows the total energy difference Δ*E* = *E*_FM_ − *E*_AFM_ as a function of Co doping *x*, assuming a random distribution of Co dopants over all Fe sites. The FM-to-AFM transition occurs at the critical doping level *x*_c_ ~ 0.2 (Fig. 3a). The estimated *x*_c_ as well as *M*_sat_ from calculations (Fig. 3b, c) is in reasonable agreement with experiments. These results indicate that the evolution of the magnetic phase with Co doping is well captured by first-principles calculations.

Detailed band structure calculations reveal that Co doping affects significantly the density-of-states (DOS) near the Fermi level. In the nonmagnetic calculations, we found a strong DOS peak in the vicinity of the Fermi level (Supplementary Fig. S9). The resulting strong Stoner instability favors the ferromagnetism within the layer, and the ferromagnetically aligned moment at each layer can be treated as a single localized Heisenberg spin, coupled through the interlayer coupling. This interlayer coupling is, however, determined by a subtle balance of pair exchange interactions between Fe/Co atoms across the vdW gap, which is sensitive to the details in the states at the Fermi level. Using total energy calculations on various interlayer spin configurations (Supplementary Fig. S10) and comparing with the classical Heisenberg Hamiltonian, we extracted the interlayer exchange interaction *J*_inter_ depending on the layer separation (*d*). We found the oscillatory behavior of *J*_inter_(*d*), well described by the RKKY model^40^ (Fig. 3d). The systematic changes of *J*_inter_(*d*) with Co doping, particularly between the nearest-neighboring layers, determine the stability of the AFM phase. These results contrast to the case of insulating vdW antiferromagnets, in which the changes in the interlayer coupling from the FM to AFM types require the stacking modifications^9–14^, and manifest the important role of conduction electrons to control the spin configurations of (Fe_1 − *x*_Co_*x*_)_4_GeTe_2_.

### Electrical detection of spin states

**Fig. 4:**
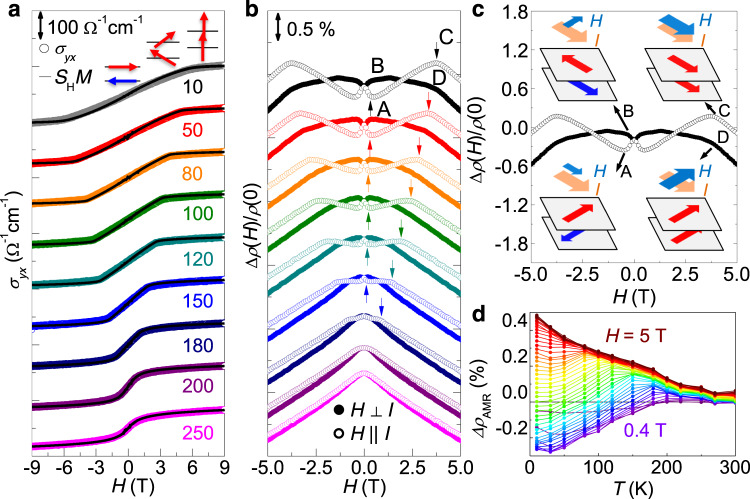
Electrical detection of the spin states. **a** The transverse conductivity *σ*_*yx*_(*H*) as a function of magnetic fields along the *c*-axis, taken at various temperatures. The *σ*_*yx*_(*H*) data are nicely reproduced by the field-dependent magnetization *M*(*H*) (black solid line) with a scaling factor *S*_H_ ≈ 0.3 V^−1^, following the linear relation of *σ*_*yx*_(*H*) = *S*_H_*M*(*H*). **b** Magnetoresistance Δ*ρ*(*H*)/*ρ*(0) under in-plane magnetic fields *H*, parallel (open) or perpendicular (solid) to the current *I* along the *a*-axis. The low field spin–flop transition field ${H}_{{\rm{sf}}}^{ab}$ and the high field saturation field ${H}_{{\rm{sat}}}^{ab}$ are indicated by the arrows. **c** Spin configurations with different relative orientations of the magnetic field *H* and the current *I*. At low *H*, the antiferromagnetically coupled spins are aligned perpendicular to *H*, either *H*∥*I* (**a**) or *H*⊥*I* (**b**). At high *H*, the saturated spins are aligned parallel to *H*, either *H*∥*I* (**c**) or *H*⊥*I* (**d**). **d** Anisotropic magnetoresistance Δ*ρ*_AMR_ as a function of temperature and in-plane magnetic field. The low-field and high-field AMR, determined by the relative orientation of Neel vector and the saturated magnetization against the current direction, respectively, which results in a sign change.

**Fig. 5:**
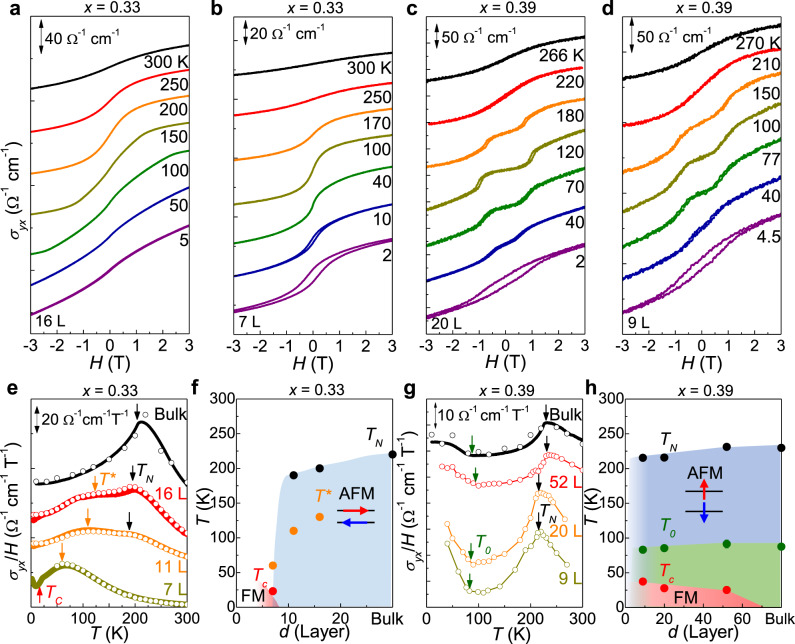
Thickness-dependent magnetic phase diagram. **a**–**d** Magnetic field-dependent transverse conductivity *σ*_*yx*_(*H*) at various temperatures of (Fe_1 − *x*_Co_*x*_)_4_GeTe_2_ nanoflakes (*x* = 0.33) for *H*∥*c* in 16L (**a**), 7L nanoflakes (**b**), and (Fe_1 − *x*_Co_*x*_)_4_GeTe_2_ nanoflakes (*x* = 0.39) for *H*∥*c* in 20L (**c**) and 9L nanoflakes (**d**). The *σ*_*yx*_(*H*) curves at low temperatures show typical coercivity in FM phases (**b**–**d**). **e** Temperature dependence of the magnetic susceptibility *χ*^*c*^(*T*) for (Fe_1 − *x*_Co_*x*_)_4_GeTe_2_ crystal (*x* = 0.33), estimated from the low-field slope of *σ*_*yx*_(*H*) at each temperature (open circle) or from the difference between *σ*_*yx*_(*T*) curves taken at *H* = ±0.1 T for *H*∥*c* (solid circle). Magnetic transition temperature *T*_N_ and *T*_C_ are indicated by the arrows, together with the characteristic temperature T*, determined by a broad hump in *χ*^*c*^(*T*). **f** Thickness-dependent magnetic phase diagram of (Fe_1 − *x*_Co_*x*_)_4_GeTe_2_ crystal (*x* = 0.33) with characteristic temperatures of *T*_N_, T*, and *T*_c_. **g** Temperature dependence of the magnetic susceptibility *χ*^*c*^(*T*) for (Fe_1 − *x*_Co_*x*_)_4_GeTe_2_ crystal (*x* = 0.39) (open circle), bulk magnetization with *H* = 0.1 T for *H*∥*c* (solid symbol). Characteristic temperature *T*_0_ is indicated by the arrows at the inflection of *χ*^*c*^(*T*) at low temperature. **h** Thickness-dependent magnetic phase diagram of (Fe_1 − *x*_Co_*x*_)_4_GeTe_2_ crystal (*x* = 0.39) with characteristic temperatures of *T*_N_, *T*_0_, and *T*_c_. The errors in the experimental data are smaller than the size of the points.

Conduction electrons are also important to probe the spin state of (Fe,Co)_4_GeTe_2_. With magnetic fields along the *c*-axis, normal to the preferred plane of the spin alignment, the moment at each layer of (Fe,Co)_4_GeTe_2_ gradually rotates until its mean direction is parallel to the field at ${H}_{{\rm{sat}}}^{c}$. This out-of-plane spin canting can be monitored by the anomalous Hall effect (AHE) due to sizable spin–orbit coupling in (Fe,Co)_4_GeTe_2_. As shown in Fig. 4a, the Hall resistivity *ρ*_*yx*_ is dominated by the anomalous contribution ${\rho }_{yx}^{\mathrm{A}}$, i.e., *ρ*_*yx*_ ≈ ${\rho }_{yx}^{\mathrm{A}}$, and thus the transverse conductivity *σ*_*yx*_ = *ρ*_*yx*_/(${\rho }_{xx}^{2}$ + ${\rho }_{yx}^{2}$) with a variation of magnetic field is nicely scaled with *M*(*H*). Moreover, the scaling factor *S*_H_ = *σ*_*yx*_/*M* is found to be almost independent of temperature (Supplementary Fig. S5), and thus *σ*_*yx*_(*H*, *T*) can quantitatively measure the out-of-plane component of net magnetization *M*(*H*, *T*). For example, the magnetic susceptibility, estimated from *χ*^*c*^(*T*) ∝ *σ*_*yx*_(*H*)/*H*, allows for experimentally determining *T*_N_ in nanoflakes (Fig. 5).

The orientation of the staggered magnetization in the plane, i.e., the Neel vector in the plane can also be effectively tracked by the electrical conductivity. Figure 4b shows the field-dependent magnetoresistance Δ*ρ*(*H*)/*ρ*(0) of (Fe,Co)_4_GeTe_2_ crystal under different field orientations in the plane, *H*∥*I* and *H*⊥*I* against a current *I*∥*a* at various temperatures. Two characteristic fields, ${H}_{{\rm{SF}}}^{ab}$ and ${H}_{{\rm{sat}}}^{ab}$, are identified from clear kinks in both Δ*ρ*(*H*)/*ρ*(0) curves. The low field ${H}_{{\rm{SF}}}^{ab}$ corresponds to the spin–flop transition, at which the Neel vectors of all domains are fully aligned perpendicular to the in-plane field^33–38^. The Neel vector *L* is rotated by 90^∘^, from *L*⊥*I* to *L*∥*I*, by switching the in-plane magnetic field from *H*∥*I* to *H*⊥*I* (Fig. 4c). This leads to a difference in magnetoresistance due to spin–orbit coupling, which is known as the anisotropic magnetoresistance (AMR) ${{\Delta }}{\rho }_{{\rm{AMR}}}^{L}$ = *ρ*_*L*∥*I*_ − *ρ*_*L*⊥*I*_. In (Fe,Co)_4_GeTe_2_, ${{\Delta }}{\rho }_{{\rm{AMR}}}^{L}$ reaches ~0.3%, comparable with other AFM metals^33–36^. With further increasing in-plane field, the moments are canted towards the field, until they are fully aligned at the saturation field ${H}_{{\rm{sat}}}^{ab}$. In this case, the AMR between the cases of *M*∥*I* or *M*⊥*I*, ${{\Delta }}{\rho }_{{\rm{AMR}}}^{M}$ = *ρ*_*M*∥*I*_ − *ρ*_*M*⊥*I*_ is also expected as found in ferromagnets^41, 42^. In (Fe,Co)_4_GeTe_2_, ${{\Delta }}{\rho }_{{\rm{AMR}}}^{L}$ and ${{\Delta }}{\rho }_{{\rm{AMR}}}^{M}$ are comparable in size and opposite in sign, inducing the sign cross of Δ*ρ*_AMR_ as summarized in Fig. 4d. Therefore, the AMR allows the electrical access to the orientation of the Neel vector or the saturated magnetization in the plane. Therefore, by combining AHE and AMR we can effectively read out the spin state of (Fe,Co)_4_GeTe_2_.

### Thickness-dependent magnetic phases

Finally, we discuss the thickness control of the magnetic state of (Fe,Co)_4_GeTe_2_. Owing to the weak vdW interlayer coupling, we obtained nanoflakes with thickness *d* down to seven layers (7L) for *x* = 0.33 and nine layers (9L) for *x* = 0.39, using mechanical exfoliation. In both cases, the in-plane resistivity *ρ*(*T*) shows the metallic behavior (~10^−3^ Ωcm) with a low-temperature upturn due to Kondo scattering, similar to the bulk case (Supplementary Figs. S12 and S13). In nanoflakes, we used the transverse conductivity *σ*_*yx*_(*H*, *T*) to monitor the field and temperature-dependent magnetization *M*(*H*, *T*), as done in the bulk case (Fig. 4a). The resulting susceptibility *χ*^*c*^(*T*) of nanoflakes (Fig. 5e, f and Supplementary Figs. S12 and S13) exhibits a clear kink, indicating the AFM transition at *T*_N_ in both cases with *x* = 0.33 and 0.39.

The detailed thickness-dependent phase diagram is different with Co doping level *x*. For nanoflakes with *x* = 0.33, *T*  decreases gradually from the bulk value with lowering thickness. In addition, in nanoflakes, we found a broad hump developed around T* in *χ*^*c*^(*T*), which shifts to low temperatures with reducing thickness (Fig. 5e). This nonmonotonous temperature dependence of *χ*^*c*^(*T*) implies that spin moments are not fully frozen by dominant AFM interaction in nanoflakes, due to additional competing FM interaction. With further reducing thickness to 7L, this competing FM interaction stabilizes the long-range FM order, evidenced by clear magnetic hysteresis of *σ*_*yx*_(*H*) with a coercive field *H*_c_ = 0.21 T at *T* = 2 K (Fig. 5b). The *H*_c_ gradually decreases with increasing temperature up to *T*_c_ ≈ 25 K (Supplementary Fig. S12). The corresponding *χ*^*c*^(*T*) exhibits no signature of AFM ordering but only a broad peak at T* ~ 40 K. The resulting phase diagram (Fig. 5g) reveals that thickness tuning offers another effective means to tune the interlayer FM and AFM interactions, even though the 2D limit is yet to be reached. It has been well established that vdW crystals expand along the *c*-axis by ~0.3–0.7%, ^43, 44^ when they are thinned to be tens of nanometer thick. Considering that the *c*-axis lattice constant shrinks by ~0.6% with Co doping of *x* = 0.39, this thinning-induced swelling affects significantly the magnetic ground state near the FM–AFM phase boundary.

In nanoflakes with higher Co doping of *x* = 0.39, located deep inside the AFM phase of the phase diagram (Fig. 3a), the AFM phase is expected to be more stable than the nanoflakes with *x* = 0.33. As shown in Fig. 5, this is indeed the case. Upon reducing the thickness down to 9L, we observed similar *σ*_*yx*_(*H*) curves with those of the bulk (Fig. 2g and Supplementary Fig. S5d). *T*_N_ is also reduced slightly to ~210 K from its bulk value of 226 K, and the low-temperature transition at *T*_0_ is also maintained without significant change. At low temperatures, we observed the magnetic hysteresis (Fig. 5c, d and Supplementary Fig. S13), indicating the FM phase, as found in the case of *x* = 0.33. The resulting thickness-dependent phase diagram for *x* = 0.39 reveals that the AFM phase in the intermediate temperature range between *T*_0_ and *T*_N_ is stable in nanoflakes. The spin–flop transition field *H*_SF_, however, is systematically reduced with lowering thickness (Supplementary Fig. S13f). These results clearly demonstrate that the stability of the AFM phase and the magnetic anisotropy can be controlled by thickness and doping levels in (Fe_1 − *x*_Co_*x*_)_4_GeTe_2_.

## Discussion

Our observations unequivocally show that (Fe,Co)_4_GeTe_2_ is an intrinsic high-*T*_N_ AFM multilayers. Although its *T*_N_ is still below room temperature, an order of magnitude enhancement of *T*_N_, as compared to other metallic vdW antiferromagnets^17–20^, is achieved by tuning the vdW interlayer coupling between the strongly FM layers of Fe_4_GeTe_2_. This approach can be applied to other recently discovered high-*T*_c_ vdW ferromagnets^26–28, 42^ to possibly realize the room temperature antiferromagnetism. Furthermore, (Fe_1 − *x*_Co_*x*_)_4_GeTe_2_ hosts at least four different spin states in terms of interlayer spin configurations (FM and AFM) and spin orientations (in-plane and out-of-plane) while keeping the same stacking structure (Fig. 3a). The switching between these states, demonstrated using doping, magnetic field, and thickness control, can be more effectively achieved in vdW heterostructures through, e.g., the exchange-spring effect^45, 46^ with an adjacent FM vdW layer or the current-induced spin–orbit torque^34, 37^ with large SOC materials, as done in metal spintronics. The intimate coupling of the spin states to electrical conduction in (Fe, Co)_4_GeTe_2_ (Fig. 4a, b) ensures the electrical readout of the spin state. These strong controllability and readability on the spin states are the manifestation of itinerant magnetism, highly distinct from insulating vdW magnets. We, therefore, envision that metallic vdW antiferromagnets, including (Fe,Co)_4_GeTe_2_ in this work, will enrich the material candidates and the spin functionalities for all vdW material-based spintronics.

## Methods

### Single-crystal growth and characterization

Single crystals were grown by a chemical vapor transport method using pre-synthesized polycrystalline sample and iodine as a transport agent. The obtained single crystals had plate-like shape with a typical size of ~1 × 1 × 0.04 mm^3^. The high crystallinity of single crystals was confirmed by X-ray diffraction (Supplementary Fig. S1). From the energy-dispersive spectroscopy measurements, we confirmed a systematic variation of Co doping *x*, in the presence of Te deficiency by ~10% and excess of the total (Fe, Co) content by ~5%, similar to pristine Fe_4_GeTe_2_^26^. Magnetization was measured under magnetic field along the *c*-axis or *ab*-plane using a superconducting quantum interference device magnetometer (MPMS, Quantum Design) and vibrating sample magnetometer option of Physical Property Measurement System (PPMS-14T, Quantum Design). The in-plane resistivity and the Hall resistivity were measured in the standard six probe configuration using a Physical Property Measurement System (PPMS-9T, Quantum Design).

### Device fabrication

Using mechanical exfoliation in the inert argon atmosphere (H_2_O < 0.1 p.p.m., O_2_ < 0.1 p.p.m.), we obtained nanoflakes of (Fe,Co)_4_GeTe_2_ on top of Si/SiO_2_ substrate, pre-treated by oxygen plasma (O_2_ = 10 s.c.c.m., *P* ~ 100 mTorr) for 5 min to remove adsorbates on the surface. The exfoliated crystal is then subsequently covered by a thin h-BN flake to prevent oxidation during device fabrication. Typically (Fe,Co)_4_GeTe_2_ flakes are of several tens of μm^2^ in the area and down to ~7 nm (~7L) in thickness, estimated by the atomic force microscopy measurements (Supplementary Fig. S11). To make electrodes for transport measurements, we employed an electron beam lithography technique, using poly(methyl methacrylate)-positive resist layer, which was spin-coated and dried in vacuum at room temperature. After etching, the patterned area of the covered h-BN flake with CF_4_ plasma, Cr(10 nm)/Au(50 nm), is deposited on the exposed surface of the nanoflake.

### STEM and EELS

STEM samples were made along [1 1 0] projections, which show the most distinguishable atomic structures. Samples were prepared with dual-beam focused ion beam systems (Helios and Helios G3, FEI). Different acceleration voltage conditions from 30 to 1 keV were used to make a thin sample with less damages. The subsequent Ar ion milling process was performed with low energy (PIPS II, Gatan Inc., USA). The atomic structure was observed using a STEM (JEM-ARM200F, JEOL, Japan) at 200 kV equipped with a fifth-order probe corrector (ASCOR, CEOS GmbH, Germany) at Materials Imaging & Analysis Center of POSTECH in Republic of Korea. The electron probe size was ~0.8 Å, and the high-angle annular dark-field detector angle was fixed from 68 to 280 mrad. The SADP was obtained using a TEM (JEM-2100F, JEOL, Japan) at 200 kV equipped with a spherical aberration corrector (CEOS GmbH, Germany). The raw STEM images were compensated with ten slices by SmartAlign and processed using a band-pass difference filter with a local window to reduce background noise (SmartAlign and Filters Pro, HREM Research Inc., Japan). For EELS analysis, we utilized another STEM (JEM-2100F, JEOL) with a spherical aberration corrector (CEOS GmbH) equipped with an EEL spectrometer (GIF Quantum ER, Gatan, USA). The used probe size was ~1.0 Å under 200 kV and the chemical analysis was performed by using a Spectrum Image via the STEM mode. The obtained spectral data from a spectral image were filtered to intensify the Fe-, Co-, and Te-edge signals by MSA (Multivariate Statistical Analysis, HREM Research Inc., Japan).

### Scanning tunneling microscopy

The surface of (Fe,Co)_4_GeTe_2_ was characterized by the scanning tunneling microscopy (STM) on a single crystal. The single crystal is first cleaved in a high vacuum (~1 × 10^−8^ torr) to obtain the clean surface and then is transferred to ultra-high vacuum (~1 × 10^−11^ torr) for the STM measurements at 77 K.

### First-principles calculations

Electronic structure calculations were performed using a full-potential linearized augmented plane wave method, implemented in WIEN2K package ^47^. Experimental lattice constants of (Fe,Co)_4_GeTe_2_ are used and exchange-correlation potential is chosen to the generalized gradient approximation of Perdew–Berke–Ernzerhof^48^. Assuming that Co is doped at all Fe sites homogeneously throughout the Fe_4_GeTe_2_ layer, the virtual crystal approximation is considered in the DFT calculation of (Fe,Co)_4_GeTe_2_.

### Soft X-ray scattering

Resonant soft X-ray scattering experiment was carried out in 6A MPK MeXiM beamline, PAL. The single crystals were cleaved in the air and thereafter immediately transferred to the chamber with the ultra-high vacuum pressure of ~8 × 10^−9^ torr. Photon energy was selected near the Fe L3-edge absorption energy value at 707.5 eV, because *q* = (0 0 3/2) peak has a different energy profile with (0 0 3) Bragg peak.

## Supplementary information

Supplementary Information

Peer Review File

## Data Availability

The data that support the findings of this study are available from the corresponding authors on request.
