# Supplementary material for: Tunable high-temperature itinerant antiferromagnetism in a van der Waals magnet
Source: Nat Commun. 2021 May 14;12:2844. doi: 10.1038/s41467-021-23122-y (PMC8121823; doi:10.1038/s41467-021-23122-y)
Supplement: Supplementary file 1 — Supplementary Information [file 41467_2021_23122_MOESM1_ESM.pdf]

Supplementary Materials: Tunable high-temperature itinerant antiferromagnetism in a van der Waals magnet

Junho Seo,[1,2,*] Eun Su An,[1,2,*] Taesu Park,[3,*] Soo-Yoon Hwang,[4] Gi-Yeop Kim,[4] Kyung Song,[5] Woo-suk Noh,[6] J. Y. Kim,[1] Gyu Seung Choi,[1,2] Minhyuk Choi,[1,2] Eunseok Oh,[1,2] Kenji Watanabe,[7] Takashi Taniguchi,[8] J. -H. Park,[2,6] Youn Jung Jo,[9] Han Woong Yeom,[1,2] Si-Young Choi,[4,†] Ji Hoon Shim,[2,3,‡] and Jun Sung Kim[1,2,§]

[1] *Center for Artificial Low Dimensional Electronic Systems, Institute for Basic Science (IBS), Pohang 37673, Korea*

[2] *Department of Physics, Pohang University of Science and Technology (POSTECH), Pohang 37673, Korea*

[3] *Department of Chemistry, Pohang University of Science and Technology (POSTECH), Pohang 37673, Korea*

[4] *Department of Materials Science and Engineering, Pohang University of Science and Technology (POSTECH), Pohang 37673, Korea*

[5] *Materials Modeling and Characterization Department, KIMS, Changwon 51508, Korea*

[6] *MPPC-CPM, Max Planck POSTECH/Korea Research Initiative, Pohang 37673, Korea*

[7] *Research Center for Functional Materials, National Institute for Materials Science, 1-1 Namiki, Tsukuba 305-0044, Japan*

[8] *International Center for Materials Nanoarchitectonics, National Institute for Materials Science, 1-1 Namiki, Tsukuba 305-0044, Japan*

[9] *Department of Physics, Kyungpook National University, Daegu 41566, Korea*

(Dated: April 9, 2021)

* equal contribution
† youngchoi@postech.ac.kr
‡ jhshim@postech.ac.kr
§ js.kim@postech.ac.kr

Supplementary Note 1. Single crystal growth

In order to grow single crystals of $(Fe,Co)_4GeTe_2$, we first synthesize polycrystalline samples using solid state reaction method. Because the stoichiometry ratio of Fe:Ge:Te = 5:1:2 in the starting mixture was found to be optimal to produce single phase polycrystalline Fe_4GeTe_2 [1], mixtures of Fe(99.998 %), Co(99.99 %), Ge(99.999 %) and Te(99.999 %) in the molar ratio of 5-x_n: x: 1: 2 ($0.25 \leq x_n \leq 1.5$) were placed in an evacuated quartz tube and then heated at 725°C for ten days. The chemical vapor transport method with iodine as a transport agent was employed for single crystal growth using the pre-synthesized polycrystalline sample. From the energy dispersive spectroscopy measurements, we confirmed a systematic variation of Co doping x, together with Te deficiency by $\sim$ 10% and $\sim$ 5% excess of the total (Fe, Co) content, similar to pristine Fe_4GeTe_2 [1]. The high crytallinity of polycrystalline ans single-crystalline samples was also confirmed by X-ray diffraction (Fig. S1a and S1d) and scanning transmission electron microscopy (Fig. 1 of the main text). All the Co-doped samples exhibit almost the same patterns with a systematic shift of Bragg peaks, for example, the (-101) peak (Fig. S1b). This indicates that $(Fe,Co)_4GeTe_2$ has the same rhombohedral structure ($R\overline{3}m$) with the pristine Fe_4GeTe_2 [1]. The lattice parameters along the a and c axes in the hexagonal representation are presented in Fig. S1c. As compared to the pristine Fe_4GeTe_2, both a and c parameters decrease with Co doping by $\sim$ 0.6% for $x = 0.39$.

Supplementary Note 2. Magnetic properties of bulk $(Fe_{1-x}Co_x)_4GeTe_2$

Figure S2 shows temperature dependent susceptibility $\chi(T)$ of the crystals with different Co doping levels (x). For $x = 0$, the ferromagnetic (FM) transition at $T_c = 270$ K is followed by the spin-reorientation transition from the easy-plane (ab) to easy-axis (c) spin alignments at $T_{\rm SR} = 110$ K. This spin-reorientation transition is quickly suppressed by Co doping, whereas the FM transition is maintained, showing the nearly constant T_c for $0 \leq x \leq 0.23$ (Figs. S2b-S2e). At the critical doping level $x = 0.26$, in addition to the FM transition, the antiferromagnetic (AFM) transition occurs at low temperatures, producing a clear kink in $\chi(T)$ at the Neel temperature $T_N = 155$ K (Fig. S2f). Upon further Co doping,

the AFM phase becomes dominant, and T_N is enhanced up to $T_N \sim 226$ K for $x \geq 0.3$ (Figs. S2g-S2i), as summarized in Fig. 3a of the main text. Moreover, for $x = 0.39$, *i.e* the maximum doping level in this work, we found another unknown magnetic transition at $T =$ 90 K, resulting in an increase of $\chi(T)$ at low temperatures. The first principles calculations well reproduce the FM-to-AFM phase transition with Co doping (Fig. 3c of the main text). The most stable AFM phase is found to be the so-called A-type, in which the FM layers are antiferromagnetically coupled across the van der Waals (vdW) gap. The dominant magnetic interaction is then expected to be FM within the layers, which is consistent with the positive Curie-Weiss temperature, obtained from the inverse susceptibility $1/\chi(T)$ (black symbols) and its Curie-Weiss fit (black dashed lines) for $x = 0.3$ - 0.39 (Figs. S3g-S3i).

The magnetic susceptibility peak has been observed at the transition to the spin cluster or spin-glass phase, which can be misinterpreted as a signature of the AFM phase transition. In order to rule out the possibility of the spin-cluster or spin-glass state, we measured the temperature dependent magnetic susceptibility, $\chi(T)$, at different cooling procedures, zero-field cooling (ZFC) and field cooling (FC). It has been well known that for spin-cluster state, the peak of $\chi(T)$ is observed at T_c, together with a pronounced bifurcation between ZFC and FC curves, which is taken as a signature of the spin-cluster state. In the $(Fe_{1-x}Co_x)_4GeTe_2$ single crystal with $x = 0.33$, we observed negligible difference between the $\chi(T)$ curves taken during ZFC and FC in both $H \parallel c$ and $H \parallel ab$ as shown in Fig. 2 of the main text and in Supplemenatary Fig. S2. These results indicate that the peak in $\chi(T)$ for the crystal with $x = 0.33$ reflects the transition, not to the spin-cluster phase, but to the antiferromagnetic (AFM) phase.

Consistently we also observed no signature of the relaxation behavior, one of the hallmark of the spin cluster state. In the spin cluster phase the change of magnetization requires a sizable time, leading to the time-dependent magnetization phenomena. For example, in the FM phase in $(Fe_{1-x}Co_x)_4GeTe_2$ crystals with low $x = 0.07$, we found a relaxation behavior as shown in Fig. S4. Once the sample is cooled down to 10 K under the in-plane field of 100 Oe and waited for $t_w \sim 200$ sec (Fig. S4b) or ~ 1000 sec (Fig. S4c), we measured the time dependent magnetization $M(t)$, right after switching off the external magnetic field ($H = 7$ Oe). The relaxation behavior of $M(t)$ is observed in both cases, which can be reproduced by the equation $M(t) = M(0) + M_1\exp[-(\mathrm{t}/\tau)^{1-\alpha}]$, where τ is the relaxation time, and α is the exponent, typically 0.4-0.6. The fit yields $\alpha \sim 0.45$ and $\tau \sim 600$ sec in both cases, which

are comparable with the spin cluster phases in, e.g. $PrRhSn_3$ and $Sr_2Mn_{0.7}Fe_{0.3}MoO_6$ [2, 3]. In contrast, for $x = 0.33$, however, we found no signature of the relaxation behavior under the similar measurement conditions, as shown in Supplementary Fig. S4. These results rule out the possibility of the spin-cluster state as an origin of the susceptibility peak in $(Fe_{1-x}Co_x)_4GeTe_2$ with $x = 0.33$. The FM-to-AFM transition with Co doping x is also confirmed by the magnetic field dependent magnetization $M(H)$. Figure S5 presents $M(H)$ curves taken at different temperatures under $H \parallel c$ (Figs. S5a-S5d), for representative cases, $x = 0.07$, 0.26, 0.33, and 0.39. The abrupt jump in the $M(H)$ curves for low x's reveals the FM phase, which eventually disappears for large x's. Instead $M(H)$ increases slowly and linearly with magnetic field for $0.26 \leq x \leq 0.33$, until it is saturated at higher magnetic field above H_{sat}, as typically found in the easy-plane antiferromagnets. For $x = 0.39$, the spin-flop transition occurs at $H \sim 1.3$ T at 100 K, but it becomes weaker below the unknown transition at 90 K. Accordingly, the saturation field H_{sat} for $H \parallel c$ is suddenly enhanced at the critical doping level $x_c \sim 0.26$ (Fig. S5e), indicating the FM-to-AFM transition with Co doping.

The different spin orientations in the AFM phases of $x = 0.33$ and 0.39 are confirmed by the distinct field-dependent magnetization $M(H)$ for $H \parallel ab$ and $H \parallel c$. For $x = 0.33$, clear kinks are observed in the $M(H)$ curve at $H_{\text{sf}} \approx \pm\, 0.2$ T only for $H \parallel ab$ not for $H \parallel c$, which are more clearly visible in the field derivative $M(H)$ curves, $dM/dH(H)$ (Fig. S6b and S6c). These are typical characters of the spin-flop transition in the easy-plane antiferromagnets, as discussed in Fig. 4 of the main text. In contrast, for $x = 0.39$, more pronounced kinks are observed for $H \parallel c$, but not for $H \parallel ab$ (Fig. S6d and S6e). This is also the finger-print of the spin-flop transition of easy-axis antiferromagnets, where the anti-parallel spins are aligned along the c axis. Below the unknown magnetic transition at $T = 90$ K, we found kinks in both $M(H)$ curves for $H \parallel ab$ and $H \parallel c$, which cannot be understood in terms of easy-plane or easy-axis AFM phases, calling for further experimental studies. Nevertheless, these results clearly demonstrate that by controlling Co doping, one can tune the types of the interlayer magnetic coupling as well as the magnetic anisotropy in $(Fe_{1-x}Co_x)_4GeTe_2$.

Finally, in order to find a more direct experimental evidence of the AFM ordering, we performed magnetic X-ray scattering experiments for the crystal with $x = 0.33$ crystal as shown in the supplementary Fig S7. In addition to the Bragg peaks at (003), a clear additional peak develops below T_N at $q = (0,0,3/2)$, which is absent at room temperature, above

T_N. The corresponding periodicity is $\sim$ 19.5 Å along the c-axis, in excellent agreement with the interlayer AFM structure, predicted by our first principles calculations. Although further studies, using neutron diffraction, are needed to determine the size and orientation of magnetic moments in the AFM phase, our magnetic X-ray scattering results provide a direct evidence of the AFM phase in $(Fe_{1-x}Co_x)_4GeTe_2$ with $x = 0.33$.

Supplementary Note 2. Transport properties of bulk $(\mathbf{Fe}_{1-x}\mathbf{Co}_x)_4\mathbf{GeTe}_2$

As shown in Fig. 2 of the main text, all the crystals exhibit a metallic behavior at high temperatures. At low temperature however they show the upturn of the resistivity at low temperatures. A rigorous distinction of metals from insulators is based on the finite resistivity extrapolated at zero temperature. Thus, in principle, metallicity can be defined, even though the resistivity increases with lowering temperatures, which is a signature of bad metal. Using the carrier density $n = \sim 6 \times 10^{27}$ m^{-3}, extracted from the contribution of the normal Hall effect and the measured resistivity, we estimated the mean free path of our crystal with $x = 0.33$ to be $\sim$ 2 nm, which is rather close to the in-plane lattice constant a 0.4 nm. Also from the quantum conductance of the 2D layers and the interlayer distance of $\sim$ 1 nm in $(Fe_{1-x}Co_x)_4GeTe_2$ crystals, we obtained a critical resistivity $\rho_c \sim 200$ $\mu\Omega$cm, which is comparable with the measured resistivity. These results show that $(Fe_{1-x}Co_x)_4GeTe_2$ crystals are in the bad metal regime.

Furthermore, at low temperatures, we found that the resistivity exhibits the -ln T dependence, followed by deviation at lower temperatures as shown in Supplementary Fig. S8 for $(Fe_{1-x}Co_x)_4GeTe_2$ crystals with $x = 0.17$, 0.23. 0.26, and 0.33. This behavior is a characteristic feature of the Kondo scattering, which has been observed in various AFM thin films with substitutional impurities with magnetic moments [4]. Recently a metastable phase of $Fe_{5-x}GeTe_2$ has been reported, which contains excess of Fe atoms at the interstitial sites above or below the Ge atoms in the unit cell [5, 6]. Considering smaller size of Co than Fe, a small amount of Co atoms can occupy these interstitial sites in our crystals, which behave as magnetic impurities. These magnetic impurities are coupled the conduction band through exchange interaction, resulting in the Kondo scattering, which becomes significant below the characteristic temperature T_K with the resistivity minimum, ρ_{min}. The excess of the resistivity $\Delta\rho(T) = \rho(T)$ - $\rho_{\mathrm{min}}(T_K)$ for $(Fe_{1-x}Co_x)_4GeTe_2$ crystals are nicely scaled as

a function of the normalized temperature by T_K, which is consistent with the prediction of Kondo scattering model as shown in Supplementary Fig. S8.

Because of this bad metal behavior, there is no significant temperature dependence of the resistivity in $(Fe_{1-x}Co_x)_4GeTe_2$ crystals. This property is useful to obtain the field dependent magnetization using the anomalous Hall effect. In Figs. S5a-S5d, we plot the transverse conductivity $\sigma_{yx} = \rho_{yx}/(\rho_{xx}^2+\rho_{yx}^2)$ as a function of magnetic field. We found that the obtained $\sigma_{yx}(H)$ curves overlap nicely with $M(H)$ data in the whole temperature range investigated in this work. The scaling factor $S_\mathrm{H} = \sigma_{yx}/M$ for different x is found to be $\sim$ 0.2-0.3 V^{-1} and almost independent of temperature (Fig. S5f). This suggests that using the transverse conductivity $\sigma_{yx}(H,T)$ measurements one can probe the out-of-plane component of net magnetization, even for nanoflakes whose magnetization is difficult to be measured using conventional methods.

Supplementary Note 3. First principles calculations

Band structures and the density of states (DOS) of nonmagnetic $(Fe_{1-x}Co_x)_4GeTe_2$ with $x = 0.0$, 0.2, and 0.5 are presented in Fig. S9. We assumed that Co atoms are doped homogeneously at the Fe sites, consistent with the electron energy loss spectroscopy (EELS) results in Fig. 1 of the main text, and thus employed virtual crystal approximation to obtain band structures and DOS. Near the Fermi level (E_F), a sharp peak is observed due to the Fe/Co d bands, which are systematically shifted to lower energies with Co doping. This DOS peak is mainly due to the relatively flat bands in the L-H symmetry line (Figs. S9a and S9b). The presence of the sharp DOS peak near the E_F promotes Stoner instability, which induces strong ferromagnetic interaction within the layer. The interlayer magnetic coupling is predicted due to band dispersion along out-of-plane direction (A-Γ, M-L, and H-K symmetry lines). With Co doping the location of the bands with different k_z dispersion changes, which results in the strong sensitivity of the interlayer magnetic interaction, depending on Co doping and also the layer distance.

In order to identify the magnetic structure of the AFM phase with large Co doping, we compared the total energies of different spin configurations for a hypothetical compound with $x = 0.5$. Since each layer of $(Fe_{1-x}Co_x)_4GeTe_2$ contains four Fe/Co sites, we tested various types of AFM structures, in terms of the intralayer and interlayer spin alignments.

As shown in Fig. S10a, the AFM phase with the lowest total energy is the A-type AFM phase (AFM-a), where ferromagnetic spin moments in the layers are antiferromagnetically coupled across the vdW gap. This is consistent with the positive Curie-Weiss temperature from the susceptibility (Figs. S3g-S3i) and the spin-flop transitions (Fig. S6). Thus Co doping mostly changes the interlayer hopping strength across the vdW gap and thus the interlayer exchange coupling, while the intralayer FM interactions remains much less affected.

The systematic changes of the interlayer exchange interaction with Co doping results in the FM-to-AFM transition as shown in Fig. 3 of the main text. The strong sensitivity of the interlayer magnetism in $(Fe_{1-x}Co_x)_4GeTe_2$, without changing stacking structure, implies the importance of conduction electrons to determine the exchange interaction across the vdW gap. We employed the classical Heisenberg Hamiltonian, describing the exchange interaction between vdW layers like

$$H_{\text{inter}} = -J_1 \sum_R S_R \cdot S_{R+d} - J_2 \sum_R S_R \cdot S_{R+2d} - J_3 \sum_R S_R \cdot S_{R+3d}, \tag{S1}$$

where S_R is a Heisenberg spin localized in the layer at position R and J_i is ith nearest layer magnetic exchange coupling constant, and d is the distance between the neighboring vdW layers. We extracted coupling constant J_i up to third-nearest layer exchange interaction from the total energy calculations of a supercell consisting of six $(Fe,Co)_4GeTe_2$ layers with different spin configurations (Fig. S10b). This verifies again that the A-type AFM phase is the most stable phase with high Co doping. As shown in Fig. 3d of the main text, the estimated J_i's are oscillating and decaying with the interlayer distance, which can be captured by the Ruderman-Kittel-Kasuya-Yosida (RKKY) type interaction, described as $J \propto 1/r^4[2k_F r\cos(2k_F r) - \sin(2k_F r)]$. Although the detailed nature of the interlayer coupling in $(Fe_{1-x}Co_x)_4GeTe_2$ cannot be understood by the simple RKKY model [7], assuming a spherical Fermi surface, this systematic change of the layer dependent $J_{\text{inter}}(d)$ with Co doping manifests the itinerant magnetism of $(Fe_{1-x}Co_x)_4GeTe_2$.

Supplementary Note 4. Scanning tunneling microscopy and atomic force microscopy

Scanning tunneling microscopy (STM) confirms the atomically flat surface of $(Fe,Co)_4GeTe_2$ crystal with a consistent in-plane lattice parameter (Fig. S11). The observed triangular

lattice corrugation corresponds to the top Te atoms, similar to those of Fe_nGeTe_2 (n = 3, 4) [1, 8]. The spacial variation of the contrast appears to be related to inhomogeneity of Co doping with a characteristic length scale of a few nm, as typically found in doped materials [9–11]. This relatively dark region covers approximately $\sim$ 32% of the total area, consistent with the Co doing concentration $x = 0.33$. Thanks to the weak vdW coupling between the layers of $(Fe,Co)_4GeTe_2$, thin flakes can be obtained using mechanical exfoliation. From the atomic force microscopy measurements, we found nanoflakes with an atomically flat surface with the typical lateral size of tens of μm^2. The thickness of these nanoflakes is typically $\sim$ 6-20 nm, corresponding to 6 to 20 layers.

Supplementary Note 5. Transport properties of $(Fe,Co)_4GeTe_2$ nanoflakes.

As shown in the Fig. 5f and 5g of main text, the magnetic properties of $(Fe,Co)_4GeTe_2$ nanoflakes change systematically with various thickness. Figures S12c-S12e show the field and temperature dependent transverse conductivity $\sigma_{yx}(H,T)$ of $(Fe,Co)_4GeTe_2$ 16 L, 11 L and 7 L respectively. As discussed above (Fig. S5), the $\sigma_{yx}(H,T)$ data is scaled with the magnetization curve $M(H,T)$ curve using a temperature independent factor S_{H}. For nanoflakes with 11L and 16L, we observed a linear field dependence of $\sigma_{yx}(H,T)$ at low magnetic fields without magnetic hysteresis, as typically found in antiferromagnets. For the 7L flake, however, a clear magnetic hysteresis appears at 2 K (Fig. S12e). The coercive field H_{c} is 0.21 T at 2 K, which gradually decreases with higher temperature and becomes negligible around $T_c \sim 25$ K (Figs. S12d and S12e). These results, together with distinct temperature dependent susceptibility (Fig. 5e in the main text), evidence the tuning the magnetic ground states between the AFM to the FM ones by thickness control in $(Fe,Co)_4GeTe_2$.

The reason why the magnetic phase of the crystal with $x = 0.33$ is sensitive to thickness is because the system is located near to the boundary between FM and AFM phases in the doping-dependent phase diagram, as shown in Fig. 3a of the main text. Thus we expect that the system with higher Co doping, located deep inside the AFM phase of the phase diagram, would be more stable than the case of $x = 0.33$. This idea is confirmed by the thickness-dependent magnetic properties of nanoflakes with $x = 0.39$. Figures S13c-S13e shows the field and temperature dependent transverse conductivity $\sigma_{yx}(H,T)$ for the nanoflakes of x = 0.39 with thickness 52 L, 20 L and 9 L. From the low-field $\sigma_{yx}(H,T)$ curves, we extracted

the temperature dependent magnetic susceptibility as shown in Fig. 5 of the main text. The clear peak at $T_N \sim 230$ K and the upturn at $T_0 \sim 80$ K are similar to the bulk case. Also, we found that the field dependent $\sigma_{yx}(H,T)$ is similar to the bulk case, clearly showing a spin-flop transition at $H_{\rm sf}$. These results confirm that the AFM phase with the out-of-plane magnetic anisotropy is maintained in the intermediate temperature range $T_0 < T < T_N$ in nanoflakes with thickness down to 9L for x =0.39. This stable AFM phase in nanoflakes with $x = 0.39$ is in strong contrast to the case of $x = 0.33$.

While the transition temperatures T_N and T_0 are not sensitive to the thickness, the spin-flop transition at $H_{\rm sf}$ strongly depends on thickness. In bulk crystals, $H_{\rm sf}(T)$ increases with lowering temperature, but eventually decrease below $\sim T_0$. This unusual temperature dependence of $H_{\rm sf}(T)$ implies that the competing FM interaction may play an important role at low temperature. Upon lowering thickness, $H_{\rm sf}$ is reduced by a factor of two in 9L-thick nanoflake. Furthermore, at low temperatures well below T_0, we observed magnetic hysteresis in $\sigma_{yx}(H,T)$ curves. While the detailed magnetic structure below T_0 is unknown at the moment, our results demonstrate that the detailed magnetic properties can be tune by controlling thickness.

---

[1] Seo, J. *et al.* Nearly room temperature ferromagnetism in a magnetic metal-rich van der Waals metal. *Sci. Adv.* **6**, eaay8912 (2020).

[2] Anand, V. K., Adroja, D. T. & Hillier, A. D. Ferromagnetic cluster spin-glass behavior in $PrRhSn_3$. *Phys. Rev. B* **85**, 014418 (2012).

[3] Wang, X. *et al.* Spin glass behavior in $Sr_2Mn_{0.7}Fe_{0.3}MoO_6$. *J. Appl. Phys.* **109**, 07C322 (2011).

[4] Khadka, D. *et al.* Kondo physics in antiferromagnetic Weyl semimetal $Mn_{3+x}Sn_{1-x}$ films. *Sci. Adv* **6**, eabc1977 (2020).

[5] Stahl, J., Shlaen, E., & Johrendt, D. The van der Waals ferromagnets $Fe_{5-\delta}GeTe_2$ and $Fe_{5-\delta-x}Ni_xGeTe_2$ - Crystal structure, stacking faults, and magnetic properties. *Z. Anorg. Allg. Chem.* **644**, 1923-1929 (2018).

[6] May, A. F. *et al.* Ferromagnetism near room temperature in the cleavable van der Waals crystal Fe_5GeTe_2. *ACS Nano* **13**, 4436-4442 (2019).

[7] Ruderman, M. A. & Kittel, C. I. Indirect exchange coupling of nuclear magnetic moments by conduction electrons. *Phys. Rev.* **96**, 99-102 (1954).

[8] Kim, K. *et al.* Large anomalous Hall current induced by topological nodal lines in a ferromagnetic van der Waals semimetal. *Nat. Mater.* **17**, 794-799 (2018).

[9] Hor, Y. S. *et al.* p-type Bi_2Se_3 for topological insulator and low-temperature thermoelectric applications. *Phys. Rev. B* **79**, 195208 (2009).

[10] Hor, Y. S. *et al.* Development of ferromagnetism in the doped topological insulator $Bi_{2-x}Mn_xTe_3$. *Phys. Rev. B* **81**, 195203 (2010).

[11] Ko, W. *et al.* Local potential fluctuation of topological surface states in $Bi_{1.5}Sb_{0.5}Te_{1.7}Se_{1.3}$ observed by Landau level spectroscopy. *Appl. Phys. Lett.* **108**, 083109 (2016).

[12] Lei, S. *et al.* High mobility in a van der Waals layered antiferromagnetic metal. *Sci. Adv.* **6**, eaay6407 (2020).

[13] McGuire, M. A. *et al.* Antiferromagnetism in the van der Waals layered spin-lozenge semiconductor $CrTe_3$. *Phys. Rev. B* **95**, 144421 (2017).

[14] Otrokov, M. M. *et al.* Prediction and observation of an antiferromagnetic topological insulator. *Nature* **576**, 416-422 (2019).

[15] Yan, J.-Q. *et al.* Evolution of structural, magnetic and transport properties in $MnBi_{2-x}Sb_xTe_4$. *Phys. Rev. B* **100**, 104409 (2019).

[16] Deng, Y. *et al.* Quantum anomalous Hall effect in intrinsic magnetic topological insulator $MnBi_2Te_4$. *Science* **367**, 895-900 (2020).

[17] Wu, J. *et al.* Natural van der Waals heterostructural single crystals with both magnetic and topological properties. *Sci. Adv.* **5**, eaax9989 (2019).

[18] Yan, J.-Q., Liu, Y. H., Parker, D., McGuire, M. A. & Sales, B. C. A-type Antiferromagnetic order in $MnBi_4Te_7$ and $MnBi_6Te_10$ single crystals. Preprint at https://arxiv.org/pdf/1910.06273.pdf (2020).

[19] Susner, M. A., Chyasnavichyus, M., McGuire, M. A., Ganesh, P. & Maksymovych, P. Metal thio- and selenophosphates as multifunctional van der Waals layered materials. *Adv. Mater.* **29**, 1602852 (2017).

[20] Kurosawa, K., Saito, S. & Yamaguchi, Y. Neutron difraction study on $MnPS_3$ and $FePS_3$. *J. Phys. Soc. Jpn* **52**, 3919-3926 (1983).

[21] Lee, J. U. *et al.* Ising-type magnetic ordering in atomically thin $FePS_3$. *Nano Lett.* **16**,

7433-7438 (2016).

[22] Wildes, A. R. *et al.* Magnetic structure of the quasi-two-dimensional antiferromagnet $NiPS_3$. *Phys. Rev. B* **92**, 224408 (2015).

[23] Kim, K. *et al.* Suppression of magnetic ordering in XXZ-type antiferromagnetic monolayer $NiPS_3$. *Nat. Commun.* **10**, 345 (2019).

[24] Wiedenmann, A., Mignod, J. R., Louisy, A., Brec, R. & Rouxel, J. Neutron difraction study of the layered compounds $MnPSe_3$ and $FePSe_3$. *Solid State Commun.* **40**, 1067-1072 (1981).

[25] Huang, B. *et al.* Layer-dependent ferromagnetism in a van der Waals crystal down to the monolayer limit. *Nature* **546**, 270-273 (2017).

[26] Wang, Z. *et al.* Determining the phase diagram of atomically thin layered antiferromagnet $CrCl_3$. *Nat. Nanotechnol.* **14**, 1116-1122 (2019).

[27] Liu, J. Y., Sun, Q., Kawazoe, Y. & Jena, P. Exfoliating biocompatible ferromagnetic Cr-trihalide monolayers. *Phys. Chem. Chem. Phys.* **18**, 8777-8784 (2016).

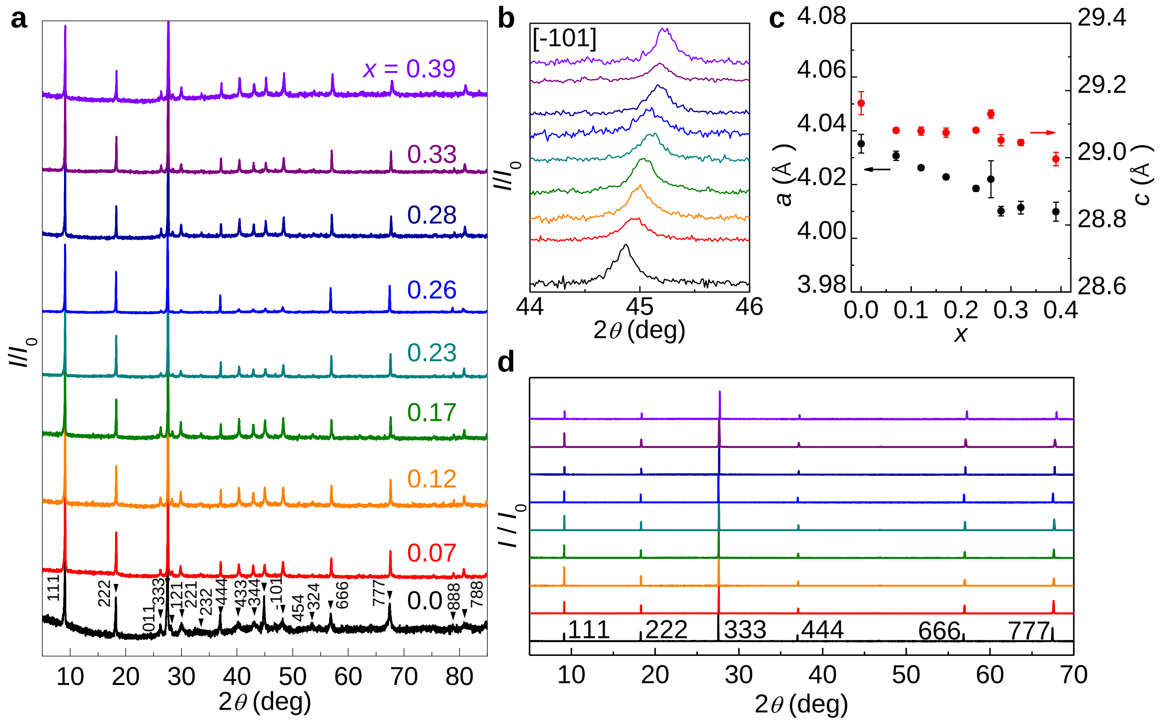

FIG. S1. **Material synthesis.** **a**, Powder X-ray diffraction patterns of $(Fe_{1-x}Co_x)_4GeTe_2$. Bragg peaks for the phase of Fe_4GeTe_2 are indicated by black triangles. **b**, Bragg peaks of (-101) for polycrystalline $(Fe_{1-x}Co_x)_4GeTe_2$. **c**, In-plane (black) and out-of-plane (red) lattice parameters obtained from Bragg peaks of Fe_4GeTe_2 phase in powder X-ray diffracation patterns of polycrystalline $(Fe_{1-x}Co_x)_4GeTe_2$. The error bar represents the standard deviation. **d**, X-ray diffraction patterns for $(Fe_{1-x}Co_x)_4GeTe_2$ ($0.0 \leq x \leq 0.39$) single crystals.

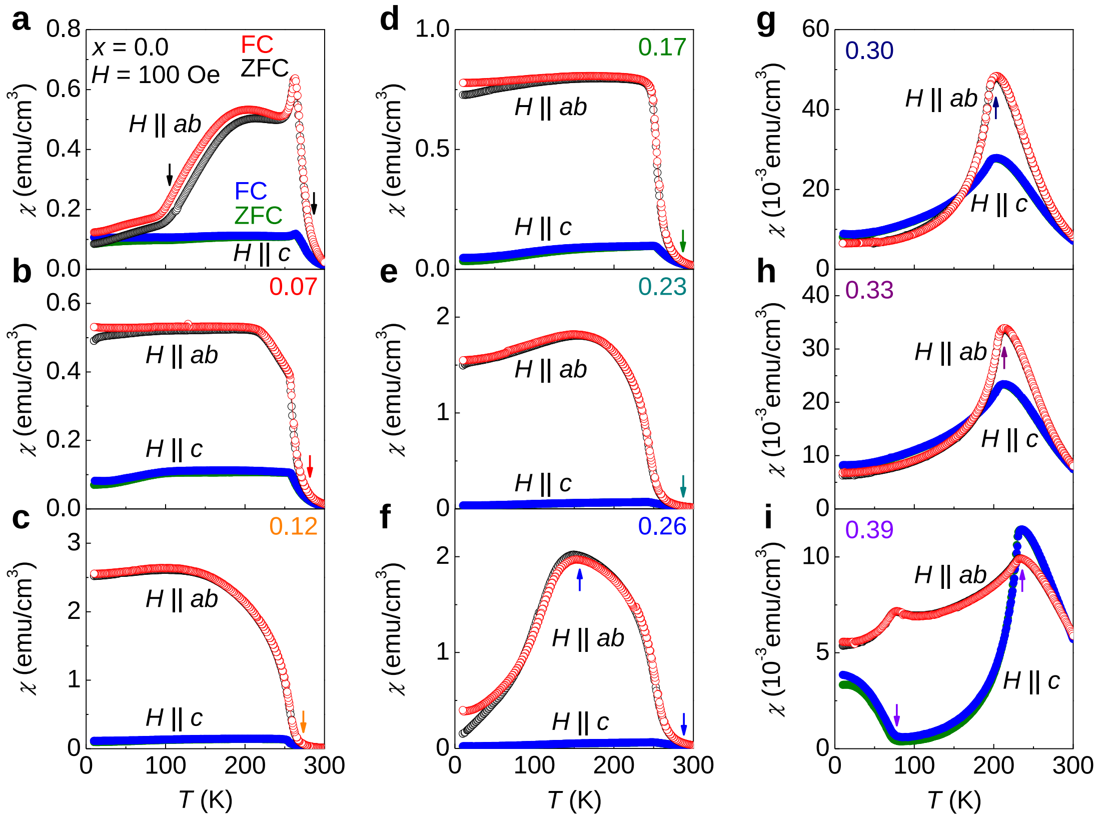

FIG. S2. **Magnetic susceptibility of $(\mathrm{Fe}_{1-x}\mathrm{Co}_x)_4\mathrm{GeTe}_2$ a-i**, Temperature dependent magnetic susceptibility $\chi(T)$ of $(\mathrm{Fe}_{1-x}\mathrm{Co}_x)_4\mathrm{GeTe}_2$ $(0.0 \leq x \leq 0.39)$, taken during zero-field cooling (ZFC) and field-cooling (FC) under $H = 100$ Oe with both magnetic field orientations, $H \parallel c$ (solid) and $H \parallel ab$ (open). Transition temperature T_c, T_N and T_{SR} in magnetic phase diagram (Fig. 3a of main text) are indicated by arrows. Negligible bifurcation between the ZFC and the FC curves are observed for $x = 0.3$ and 0.33, below T_N and for $x = 0.39$ in the intermediate temperature range $T_0 < T < T_N$, confirming the AFM phase.

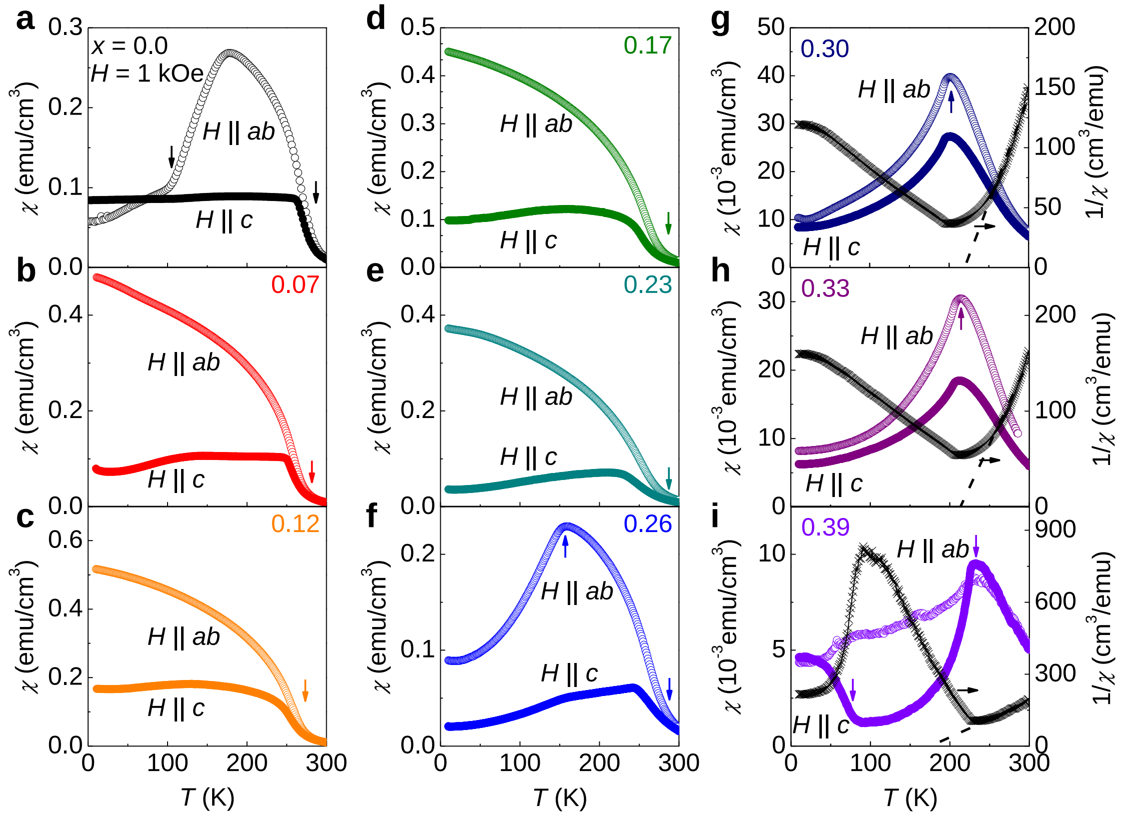

FIG. S3. **Magnetic susceptibility of $(\mathbf{Fe}_{1-x}\mathbf{Co}_x)_4\mathbf{GeTe}_2$ a-i**, Temperature dependent magnetic susceptibility $\chi(T)$ taken under $H = 1$ kOe for $H \parallel c$ (solid) and $H \parallel ab$ (open) of $(\mathrm{Fe}_{1-x}\mathrm{Co}_x)_4\mathrm{GeTe}_2$ ($0.0 \le x \le 0.39$). Transition temperature T_c, T_N, T_{SR} and T_0 in magnetic phase diagram (Fig. 3a of main text) are indicated by arrows. For antiferromagnetic ($0.30 \le x \le 0.39$) phases, inverse susceptibility for $H \parallel c$ (black symbol) and its Curie-Weiss plot (black dashed line) are also plotted (**g-i**), As mentioned in the main text, the positive Curie-Weiss temperature is observed in the AFM phase, suggesting the dominant FM interaction within the layers.

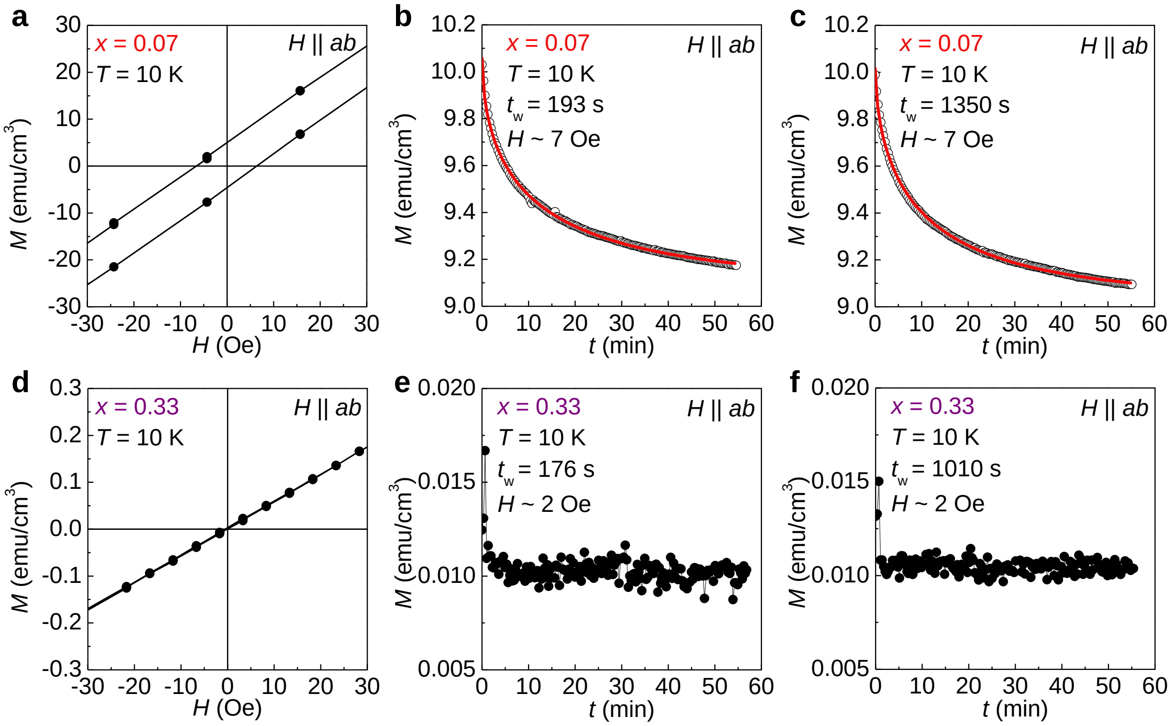

FIG. S4. **Thermoremanent magnetization relaxation in $(\mathbf{Fe}_{1-x}\mathbf{Co}_x)_4\mathbf{GeTe}_2$.** **a**, Magnetic field dependent magnetization for $x = 0.07$ at $T = 10$ K. Clear hysteresis behavior with coercive field $H_c \sim 6$ Oe is observed. **b,c**, Thermoremanent magnetization relaxation for $x = 0.07$ at $T = 10$ K. For the thermoremanent magnetization measurement, the sample was field-cooled with magnetic field $H = 100$ Oe from $T = 300$ K to 10 K then we waited for a waiting time t_{w}. Thereafter the field was reduced to almost zero and the magnetization was measured as a function of the elapsed time. The red solid line is the best fit of $M(t) = M(0) + M_1\exp[-(\mathrm{t}/\tau)^{1-\alpha}]$, where τ is the relaxation time, and α is the exponent. The fit yields $\alpha \sim 0.45$ and $\tau \sim 600$ sec in both cases. **d**, Magnetic field dependent magnetization for $x = 0.33$ at $T = 10$ K. In contrast to $x = 0.07$ case, no hysteresis behavior is observed. **e,f**, Thermoremanent magnetization relaxation for $x = 0.33$ at $T = 10$ K with waiting time t_{w}. Here, no signature of relaxation behavior is observed.

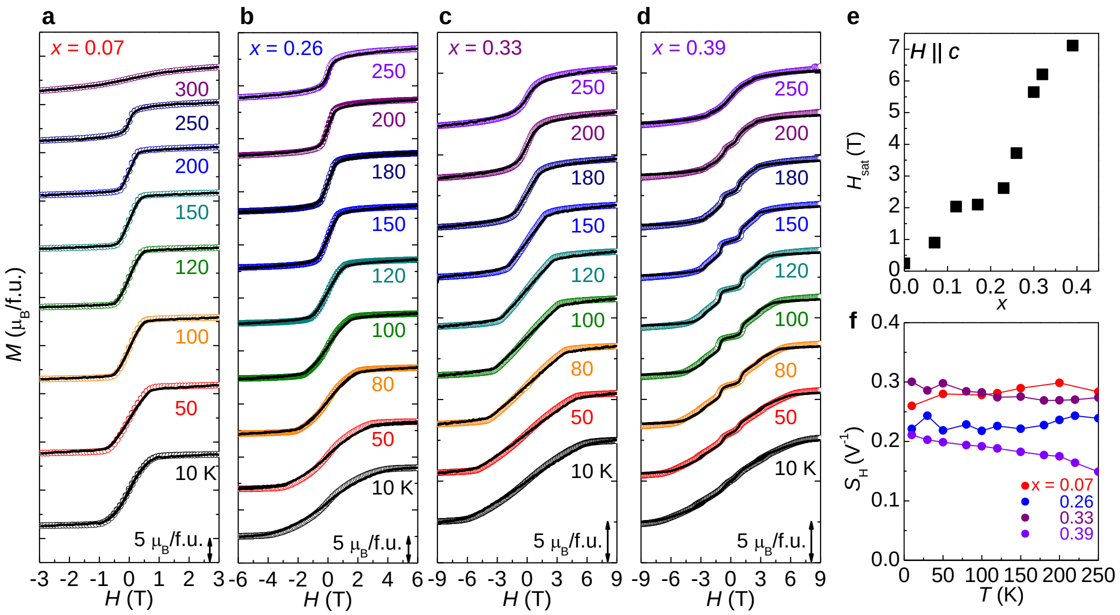

FIG. S5. **Magnetic properties and anomalous Hall effect of $(\mathbf{Fe}_{1-x}\mathbf{Co}_x)_4\mathbf{GeTe}_2$. a-d**, Magnetic field dependent magnetization $M(H)$ of $(\mathrm{Fe}_{1-x}\mathrm{Co}_x)_4\mathrm{GeTe}_2$ when $x = 0.07$ (**a**), $x = 0.26$ (**b**), $x = 0.33$ (**c**) and $x = 0.39$ (**d**) at various temperatures for $H \parallel c$. $M(H)$ curves are nicely reproduced by the field dependent Hall conductivity $\sigma_{yx}(H)$ (black solid line) with a scaling factor S_H, following the linear relation of $\sigma_{yx}(H) = S_\mathrm{H} M(H)$. **e**, The saturation fields H_sat for $H \parallel c$ as a function of Co doping x. **f**, Temperature dependence of $S_\mathrm{H} = \sigma_{yx}(H)/M(H)$ for $(\mathrm{Fe}_{1-x}\mathrm{Co}_x)_4\mathrm{GeTe}_2$ as a function of x. The errors in the experimental data are smaller than the size of the points.

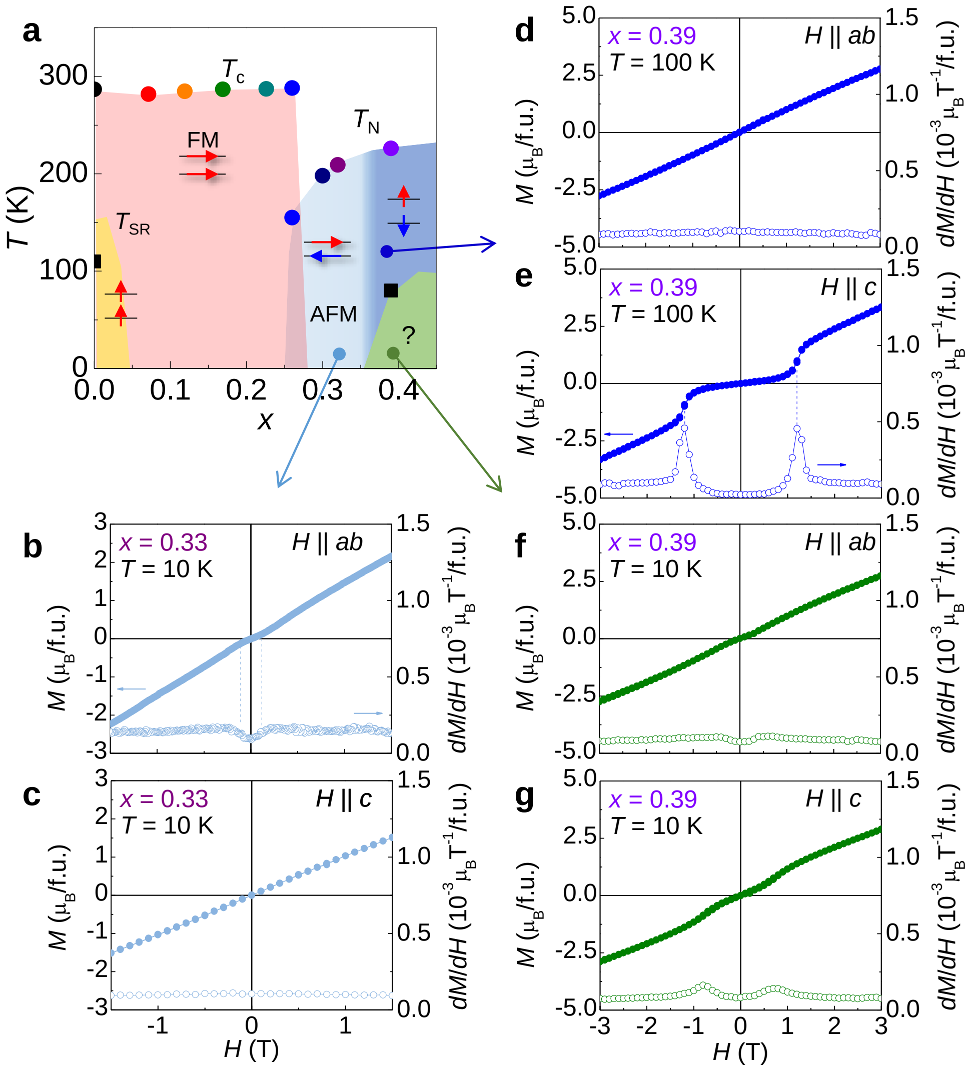

FIG. S6. **Spin flop transition in antiferromagnetic $(Fe_{1-x}Co_x)_4GeTe_2$.** **a**, Doping dependent magnetic phase diagram of $(Fe_{1-x}Co_x)_4GeTe_2$. **b-g**, Magnetization $M(H)$ (solid) and its derivative dM/dH (open) as a function of magnetic field for $x = 0.33$ at $T = 10$ K (**b,c**) and $x = 0.39$ at $T = 100$ K (**d,e**) and $T = 10$ K (**f,g**). The spin flop transitions are indicated by the vertical dashed lines. The errors in the experimental data are smaller than the size of the points.

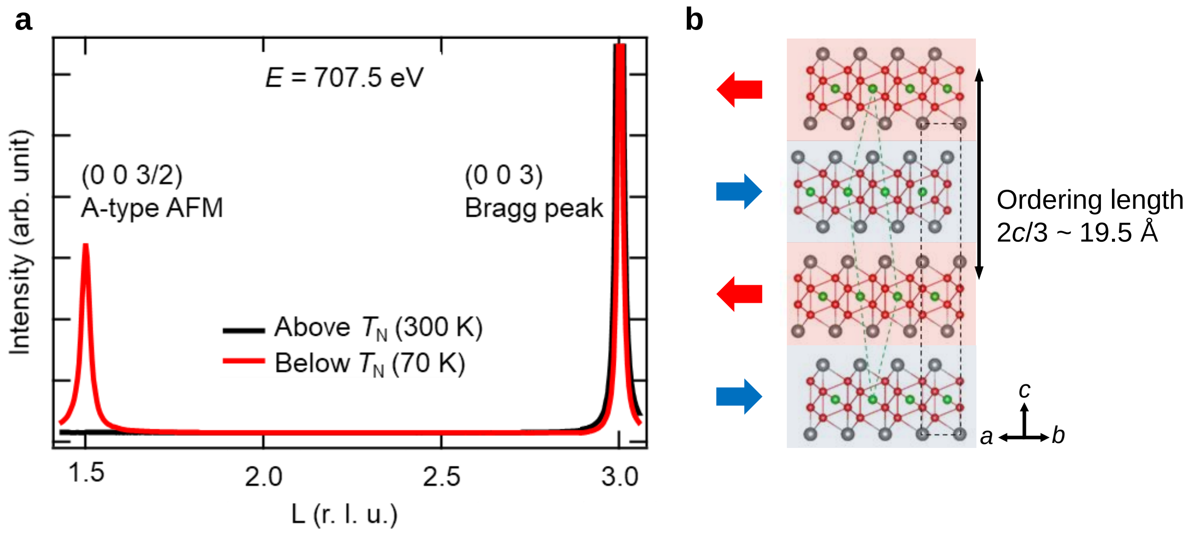

FIG. S7. **Resonant soft X-ray scattering of $(Fe_{0.67}Co_{0.33})_4GeTe_2$. a**, Resonant soft X-ray scattering intensity at 300 K ($> T_N$) and 70 K ($< T_N$). Photon energy was selected near the Fe L3-edge absorption energy value, 707.5 eV, because $q = (0\ 0\ 3/2)$ peak have different energy profile with (0 0 3) Bragg peak. In addition to Bragg peak at $q = (0\ 0\ 3)$, additional peak at $q = (0\ 0\ 3/2)$ is developed below T_N. **b**, Crystal structure of $(Fe_{0.67}Co_{0.33})_4GeTe_2$ with the interlayer AFM structure, indicated by red and blue shades. The period of 19.5 Å, corresponding to $q = (0\ 0\ 3/2)$, is consistent with the interlayer AFM phase.

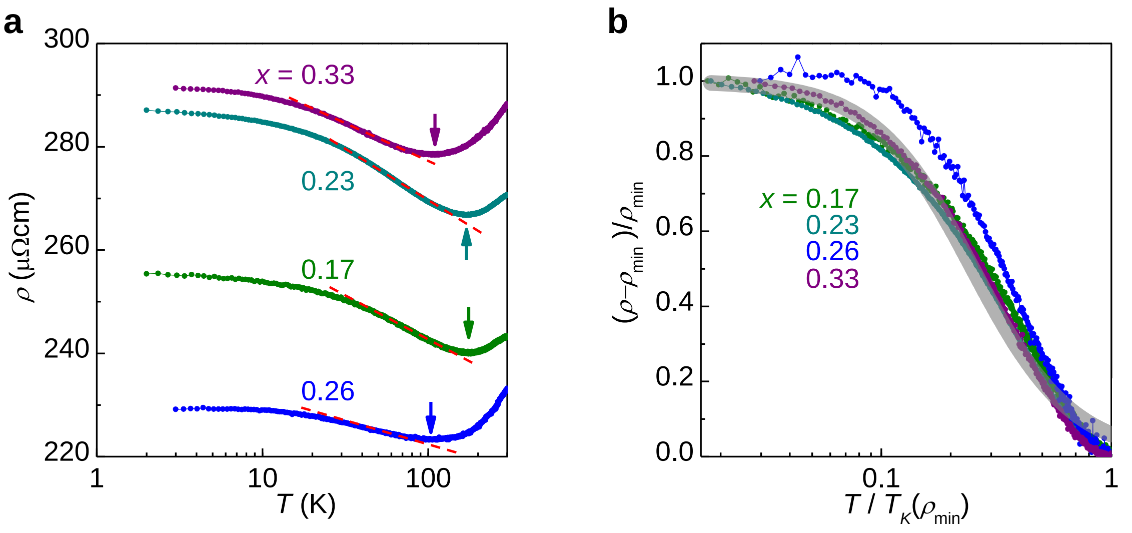

FIG. S8. **Kondo scattering in $(Fe_{1-x}Co_x)_4GeTe_2$. a**, Temperature dependent resistivity $\rho(T)$ for x = 0.17, 0.23. 0.26, and 0.33. Clear low-temperature upturn is commonly observed, which is described by -ln$T$ (dashed lines) in the intermediate temperature range. **b**, Excess of the resistivity $\Delta\rho(T) = \rho(T)$ - $\rho_{\rm min}(T_K)$ as a function of normalized temperature T/T_K, due to Kondo scattering. T_K is the characteristic temperature with the resistivity minimum $\rho_{\rm min}$. The predicted $\Delta\rho(T/T_K)$ curve (solid line) of Kondo scattering model is presented for comparison.

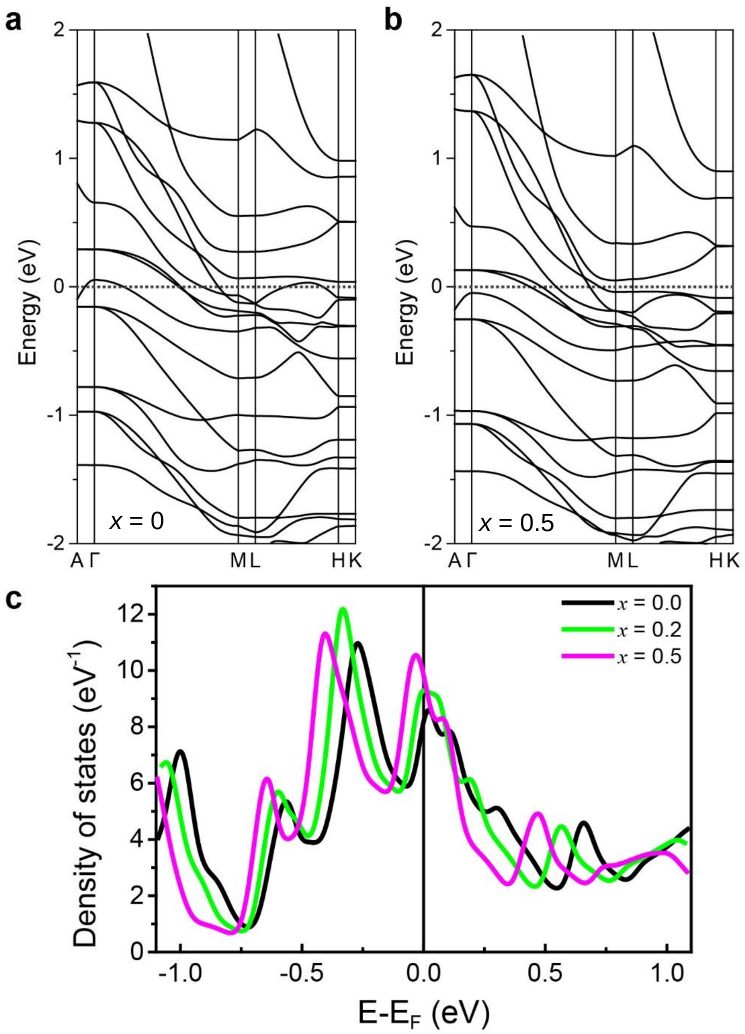

FIG. S9. **Electronic structures of $(Fe_{1-x}Co_x)_4GeTe_2$. a, b**, Calculated nonmagnetic band structures of $(Fe_{1-x}Co_x)_4GeTe_2$ for $x = 0$ (**a**) and $x = 0.5$ (**b**). **c**, Nonmagnetic density of states near Fermi level depending on Co doping level ($0 \leq x \leq 0.5$).

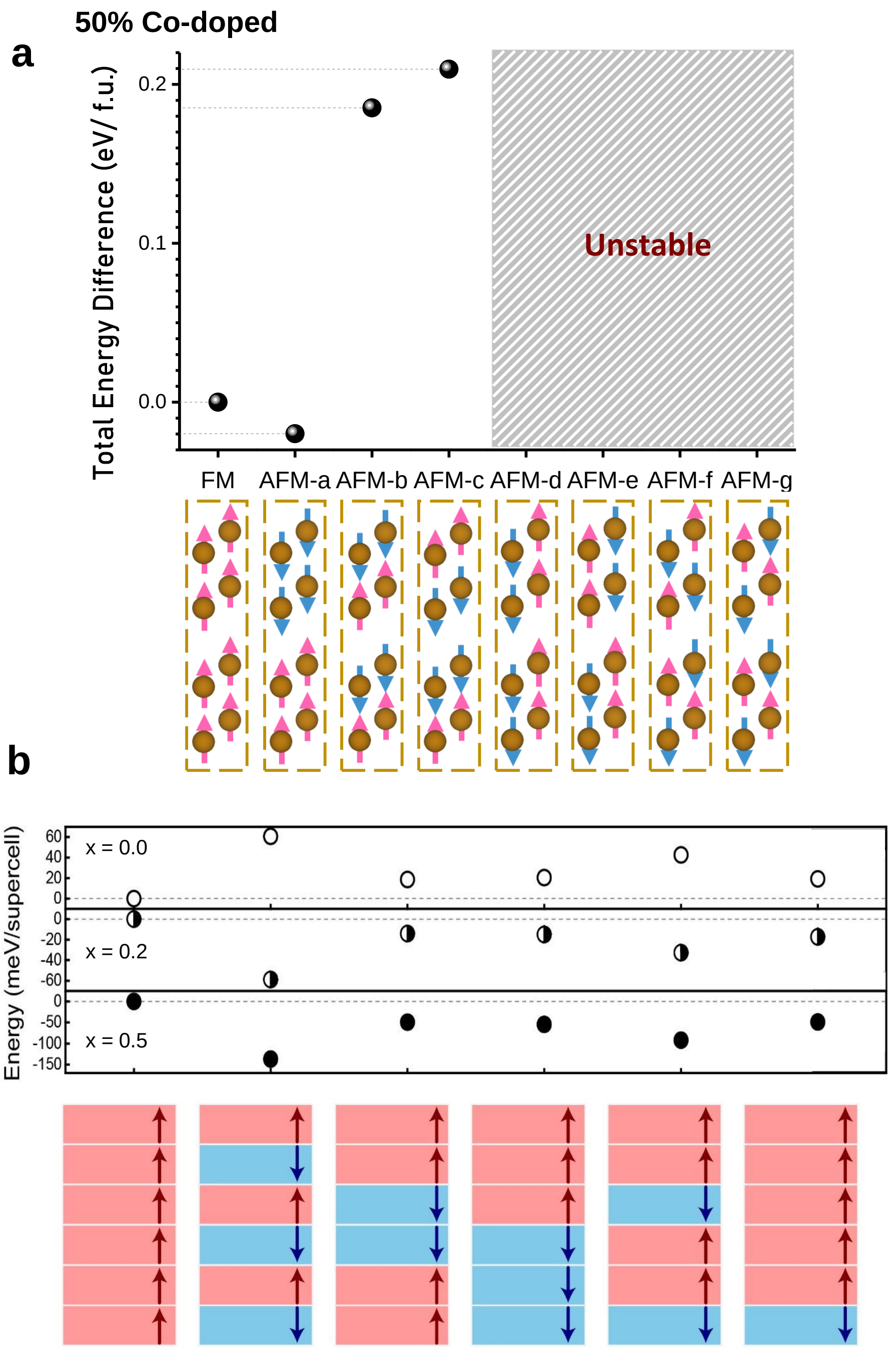

FIG. S10. **Comparison of total energies for various spin configurations of $(\mathbf{Fe}_{1-x}\mathbf{Co}_x)_4\mathbf{GeTe}_2$.** **a**, Total energy difference of various intralayer and interlayer spin configurations with respect to the A-type AFM phase (AFM-a) for $x = 0.5$. The corresponding AFM structures (AFM-a to AFM-f) are displayed below. **b**, Total energy difference of various interlayer spin configurations with respect to that of the ferromagnetic state for $x = 0.0$, 0.2 and 0.5. The corresponding supercell structures consisting of six $(Fe_{1-x}Co_x)_4GeTe_2$ layers are presented below.

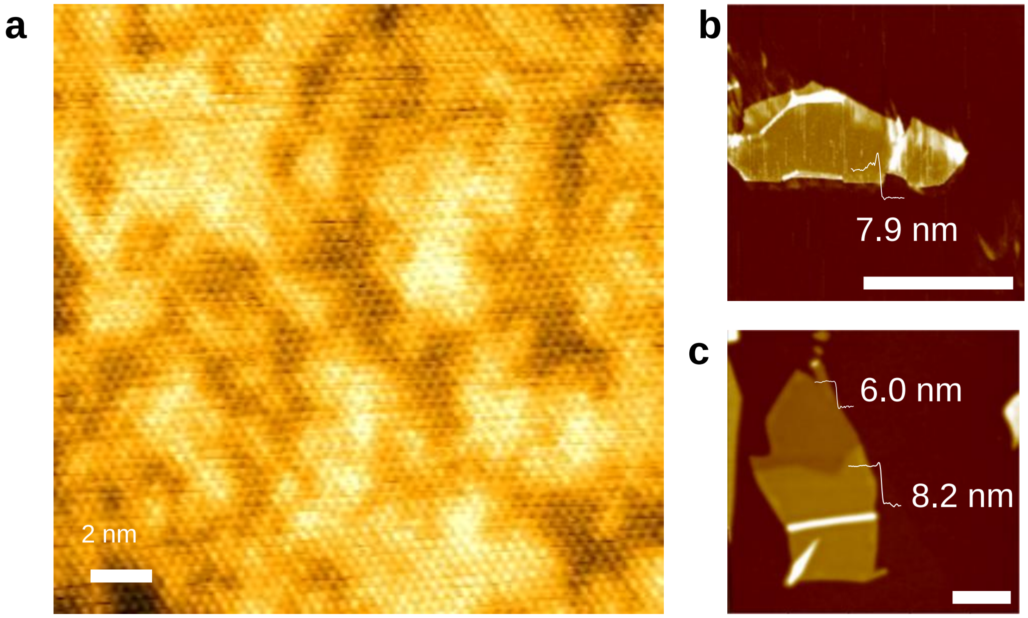

FIG. S11. **STM and AFM images of $(Fe_{1-x}Co_x)_4GeTe_2$ (x=0.33) a**, Scanning tunneling microscopy (STM) image of $(Fe,Co)_4GeTe_2$ crystal. The atomically flat surface and the triangular lattice corrugation corresponding to the top Te atoms are clearly resolved. **b,c**, Atomic force microscopy images of $(Fe,Co)_4GeTe_2$ with 3 μm scale bar. The inset shows the AFM height profile with the corresponding thickness.

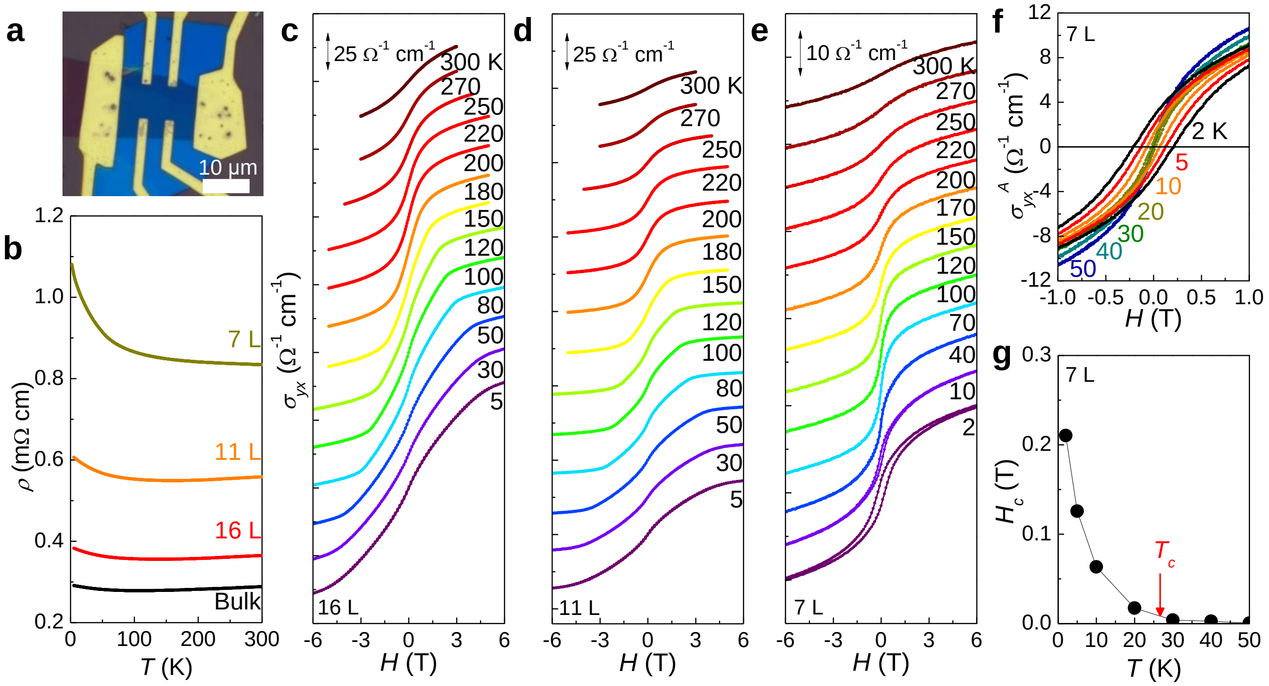

FIG. S12. **Anomalous Hall effect of $(\mathbf{Fe}_{1-x}\mathbf{Co}_x)_4\mathbf{GeTe}_2$ nanoflake.** **(x=0.33)** **a**, The optical image of BN-covered 7-layer-thick (7 L) $(Fe,Co)_4GeTe_2$ crystal with the scale bar of 10 μm. **b**, Temperature dependent in-plane resistivity $\rho(T)$ for bulk and nanoflakes with various thickness, indicated by number of the layers. **c-e**, Magnetic field dependent transverse conductivity $\sigma_{yx}(H)$ at various temperature $H \parallel c$ in 16L (**c**), 11L (**d**) and 7L nanoflakes (**e**). **f**, Temperature dependent anomalous Hall conductivity $\sigma_{yx}^A(H)$ of 7L nanoflake with magnified magnetic field range. The magnetic hysteresis is clearly observed in the field dependent $\sigma_{yx}^A(H)$ for 7L crystal, taken at $T = 2$ K. **g**, The temperature dependence of coercive field H_c for 7L crystal. The coercive field, which is $H_c = 0.21$ T at 2 K, gradually decreases with increasing temperature up to $T_c = 30$ K. The arrow indicates Curie temperature estimated by temperature dependence of coercive field. The errors in the experimental data are smaller than the size of the points.

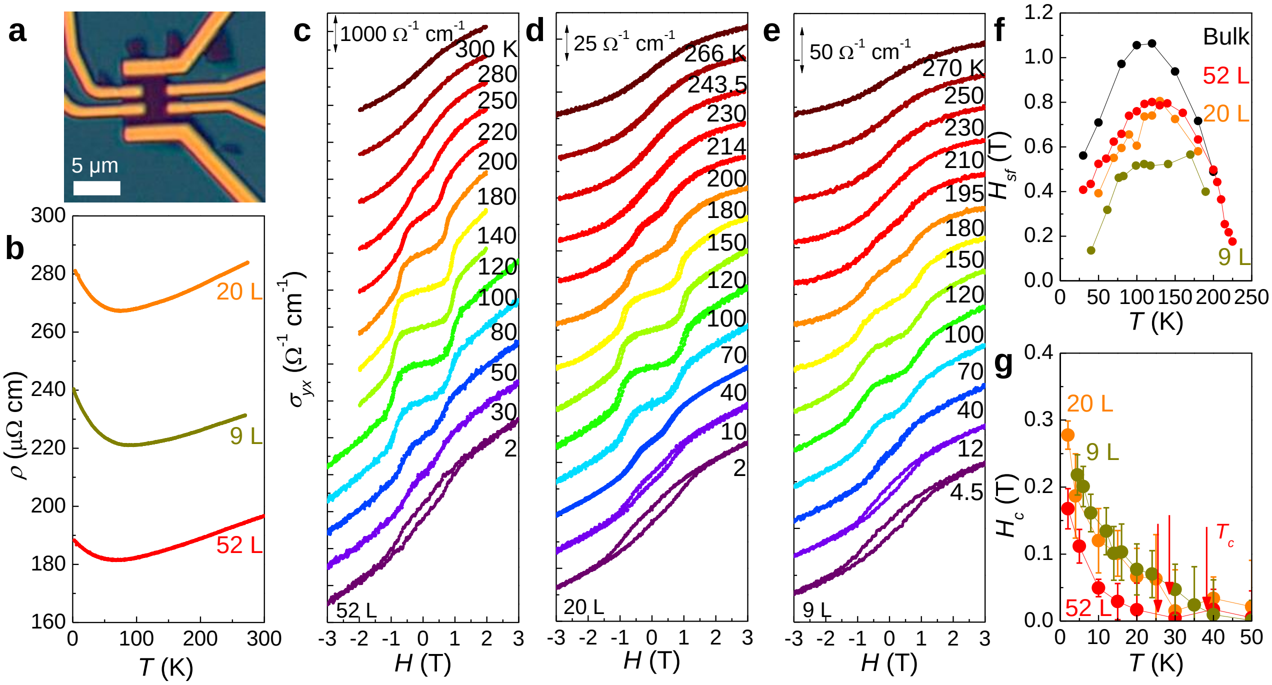

FIG. S13. **Anomalous Hall effect of $(\mathbf{Fe}_{1-x}\mathbf{Co}_x)_4\mathbf{GeTe}_2$ nanoflake.** **(x=0.39)** **a**, The optical image of BN-covered 9-layer-thick (9 L) $(Fe,Co)_4GeTe_2$ crystal with the scale bar of 5 μm. **b**, Temperature dependent in-plane resistivity $\rho(T)$ for nanoflakes with various thickness, indicated by number of the layers. **c-e**, Magnetic field dependent transverse conductivity $\sigma_{yx}(H)$ at various temperature $H \parallel c$ in 52L (**c**), 20L (**d**) and 9L nanoflakes (**e**). **f**, Temperature dependent spin-flop field $H_{\rm sf}$ for bulk and nanoflakes with various thickness, indicated by number of the layers. **g**, The temperature dependence of coercive field $H_{\rm c}$ for nanoflakes. The coercive field for nanodevices gradually decreases with increasing temperature. The arrow indicates Curie temperature estimated by temperature dependence of coercive field. The error bars represent the minimum and maximum measured values.

TABLE S1. Magnetic properties of various van der Waals antiferromagnets.

Material	Number of layers (L)	T_N (K)	M (μ_B/.f.u.)	Conductivity	Reference
$(Fe_{0.67}Co_{0.33})_4GeTe_2$	bulk	210	5.7	Metal	This work
	11	190		Metal	This work
$(Fe_{0.61}Co_{0.39})_4GeTe_2$	bulk	226	5.5	Metal	This work
	9	210		Metal	This work
$GdTe_3$	bulk	17	-	Metal	Ref. 12
	17	9.5	-		Ref. 12
$CrTe_3$	bulk	53	2.0	$E_g \sim 0.26$ eV	Ref. 13
$MnBi_2Te_4$	bulk	25	3.56	$E_g \sim 0.088$ eV	Ref. 14, 15
	3	18			Ref. 16
$MnBi_4Te_7$	bulk	13	4.16	Metal	Ref. 17, 18
$MnBi_6Te_{10}$	bulk	11	4.75	Metal	Ref. 17, 18
$MnPS_3$	bulk	82	4.1(3)	$E_g \sim 3.0$ eV	Ref. 19, 20
$FePS_3$	bulk	116	5.1(6)	$E_g \sim 1.6$ eV	Ref. 19, 20
	10	110			Ref. 21
	1	110			Ref. 21
$NiPS_3$	bulk	155	1.05	$E_g \sim 1.7$ eV	Ref. 19, 22
	15	159			Ref. 23
	2	129			Ref. 23
$MnPSe_3$	bulk	74	4.74	$E_g \sim 2.3$ eV	Ref. 19, 24
$FePSe_3$	bulk	112	4.9	$E_g \sim 1.3$ eV	Ref. 19, 24
$NiPSe_3$	bulk	206	-	-	Ref. 19
CrI_3^*	3	61			Ref. 25
	1	45			Ref. 25
$CrCl_3$	bulk	17	3	$E_g \sim 2.28$ eV	Ref. 26, 27
	13	18			Ref. 26
	2	16			Ref. 26

Note (asterisk): CrI_3 is A-type antiferromagnet when thinned down to few layers.
